# Geminivirus diversity and community structure at the scale of agro-ecological systems in two localities of Burkina Faso

**DOI:** 10.1093/ismeco/ycag196

**Published:** 2026-07-08

**Authors:** Alassane Ouattara, Sélim Ben Chéhida, Murielle Hoareau, Sohini Claverie, Daniel Houa Otron, Edgar Valentin Traoré, Oumar Traoré, Nicolas Barro, Fidèle Tiendrébéogo, Darren P Martin, Jean-Michel Lett, Pierre Lefeuvre

**Affiliations:** INERA, Ouagadougou 01 BP 476, 01, Burkina Faso; CIRAD, UMR PVBMT, St-Pierre, La Réunion F-97410, France; Université Joseph Ki-Zerbo, Ouagadougou 03 BP 7021, 03, Burkina Faso; LMI Patho-Bios, IRD-INERA, Ouagadougou 01 BP 476, 01, Burkina Faso; CIRAD, UMR PVBMT, St-Pierre, La Réunion F-97410, France; INRAE Grand-Est Colmar, UMR 1131A Santé de la Vigne et Qualité du Vin, Colmar 68000, France; CIRAD, UMR PVBMT, St-Pierre, La Réunion F-97410, France; CIRAD, UMR PVBMT, St-Pierre, La Réunion F-97410, France; Université de La Réunion, UMR PVBMT, St-Pierre, La Réunion F-97410, France; CIRAD, UMR PVBMT, St-Pierre, La Réunion F-97410, France; Université Félix Houphouët-Boigny, Abidjan 01 BP V 34, 01, Côte d’Ivoire; INERA, Ouagadougou 01 BP 476, 01, Burkina Faso; INERA, Ouagadougou 01 BP 476, 01, Burkina Faso; Laboratoire National de Biosécurité, Ouagadougou 06 BP 10798, 06, Burkina Faso; Université Joseph Ki-Zerbo, Ouagadougou 03 BP 7021, 03, Burkina Faso; INERA, Ouagadougou 01 BP 476, 01, Burkina Faso; Computational Biology Division, Department of Integrative Biomedical Sciences, Institute of Infectious Diseases and Molecular Medicine, University of Cape Town, Observatory, South Africa; CIRAD, UMR PVBMT, St-Pierre, La Réunion F-97410, France; CIRAD, UMR PVBMT, St-Pierre, La Réunion F-97410, France; CIRAD, UMR PVBMT, Can Tho University College of Agriculture, Can Tho, Viet Nam

**Keywords:** CRESS DNA viruses, virus diversity, ecological networks, recombination, plant–virus interactions

## Abstract

Circular replication-associated protein encoding single stranded (CRESS) deoxyribonucleic acid (DNA) viruses have emerged to become one of the largest and most devastating groups of crops-infecting viruses in tropical and subtropical regions of the world. In West Africa, renewed outbreaks of disease-like symptoms in the field have prompted surveys that revealed the circulation of multiple CRESS DNA virus species. Recognizing that plant viral diseases may be best understood at the ecosystem scale, we conducted a survey of plant-associated CRESS DNA virus communities in two localities of Burkina Faso (1068 plant samples). CRESS DNA viruses were detected in 41% and 13% of samples from cultivated and uncultivated plants, respectively. Virus community maps constructed from the 30 uncovered species of geminivirus and associated satellites revealed strong modularity. Whereas few co-infections involving viruses belonging to different host–virus modules were observed, evidence of recombination was nevertheless detected between viruses belonging to different modules. This suggests that co-infections between viruses in different modules does at least occasionally occur: possibly as a consequence of seasonal variations in virus–host community structures with the inter-cropping period in particular promoting such “inter-module” co-infections.

## Introduction

Circular replication-associated protein encoding single stranded (CRESS) deoxyribonucleic acid (DNA) viruses (from the *Cressdnaviricota* phylum) is a phylum of viruses infecting organisms across the eukaryotic domain [1]. CRESS DNA viruses have emerged to become one of the largest and most economically important groups of plant-infecting viruses [2], with the geminiviruses (viruses in the family *Geminiviridae*) counting as the most economically significant [3].

Geminiviruses are arthropod-transmitted plant-infecting viruses characterized by their unique twinned icosahedral virions. Geminivirus-induced diseases represent a global threat to worldwide vegetable production, particularly in tropical and subtropical regions of the world [4–6]. During the past decades, our knowledge of geminivirus diversity has been tremendously enhanced through the use of rolling circle amplification techniques that enable efficient and sequence agnostic genome amplification prior to sequencing [7]. These techniques sparked the discovery of large variations in terms of geminivirus genomic organization, associations with satellite molecules and genome component numbers. The diverse menagerie of geminiviruses that have been discovered has prompted the definition of multiple new geminivirus genera along with entirely new families to accommodate the satellite molecules that are associated with geminiviruses [8–10].

Within the family *Geminiviridae*, 15 genera are currently accepted (ICTV), the most described of which, the genus *Begomovirus*, encompasses viruses with either monopartite genomes, presenting with a single DNA-A like component of ~2.7 kb, or bipartite genomes with a DNA-A and DNA-B components, both of ~2.7 kb. These components are frequently associated with satellites of ~1.4 kb, that depend on a helper-geminivirus for transmission and, in some instances, also for replication.

Two families of geminivirus-associated satellites have been defined: the *Alphasatellitidae* (also known as alphasatellites; [10]) and the *Tolecusatellitidae* (a family that accommodate betasatellites and deltasatellites; [9]). Whereas alphasatellites encode a replication initiator protein (Rep) and are capable of self-replication, betasatellites do not and are therefore dependent on their helper-virus for replication. Although both alphasatellites and betasatellites have been associated with symptom modulation and the suppression of host antiviral defenses, their precise impacts on the pathogenicity, virulence, transmission and general evolutionary fitness of their helper viruses remain unknown [11].

Nevertheless, ensembles comprising begomoviruses and a range of different satellites have been associated with the emergence of multiple important diseases throughout the tropical and temperate regions of Europe, Asia [5, 12], and Africa [13–19].

Besides uncertainty with respect to the role(s) of satellite molecules in the etiology of geminivirus diseases, it is also uncertain how relaxed or constrained are the associations between the DNA-A and DNA-B components of bipartite geminiviruses. It is indeed apparent that, in at least some (but possibly many) circumstances, DNA-A and DNA-B interactions are malleable [20–22], with the fitness of particular combinations of DNA-A and DNA-B molecules possibly being strongly dependent on the ecological and seasonal contexts within which viruses occur.

Additionally, whereas it has been clearly established that some of the geminiviruses that currently cause major diseases likely originated from viruses that were adapted to infecting non cultivated plants [23], several of the main geminiviral threats to crops are now well adapted to their crop hosts [24]. As most crops are not grown year-round and vector species have only few month lifespans (up to two months for the begomovirus vector *Bemisia tabaci*, [25], uncultivated plants must serve as alternative hosts during months long inter-cropping periods. Therefore, even for geminiviruses that were to infect crops within relatively homogeneous and well-ordered agricultural environments, the compositions of the virus–host communities to which these viruses belong necessarily include non-crop hosts and undergo variations throughout the year. In this context, diverse opportunities for infection and viral mixing may arise. Beyond facilitating or constraining infection through synergy or competition [26, 27], such co-occurrence can promote viral evolution by increasing opportunities for genetic exchange through recombination.

It is therefore essential to understand how viruses are distributed within plant communities in natural and agricultural settings, and how these malleable interactions may influence viral persistence and shape evolutionary trajectories [23, 28, 29].

Here, we used two complementary metagenomic approaches based on high throughput sequencing following rolling circle amplification of DNA [30, 31] to investigate host–geminivirus associations at the ecosystem scale in two regions of Burkina Faso. The CRESS DNA virus community map that was built from the analysis of virus–host and virus–virus association revealed a complex associations of helper and satellites. Whereas we observed a strong modularity in the virus–host community structure, we nevertheless detect evidence of recombination exchange that indicates that mixed infections involving viruses from different community modules do at least occasionally occur and promote the general community diversity.

## Materials and methods

### Sample collection

**Figure 1:**
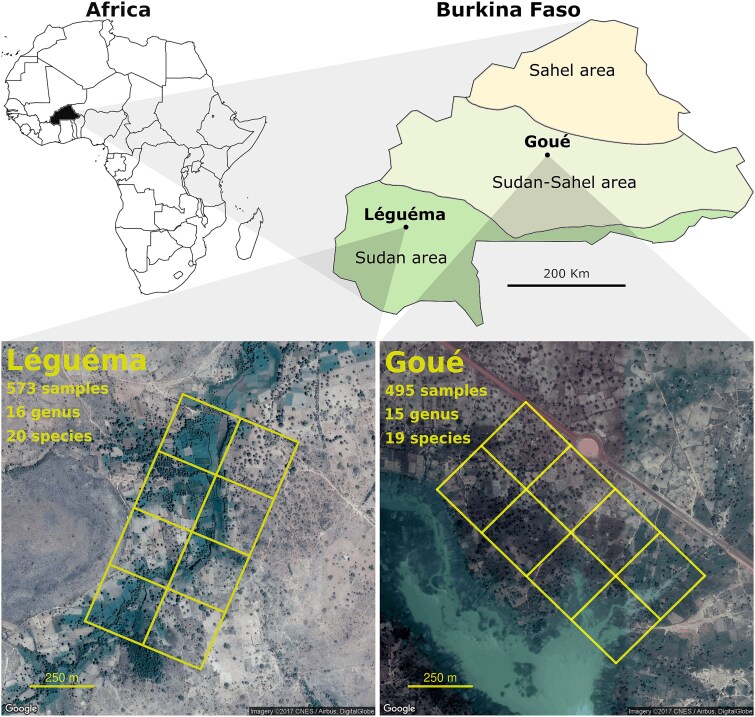
Map of locations surveyed in Burkina Faso. Each grid had dimensions of 500 × 1000 m with meshes of 250 × 250 m and covered a cultivated and uncultivated setting.

In March and April 2016, sampling campaigns were conducted in two localities of Burkina Faso (Fig. 1): Léguéma (South-West near Bobo-Dioulasso; latitude: 11.226°, longitude: −4.164°) and Goué (Central-East near Ouagadougou; latitude: 12.567°, longitude: −1.471°), respectively, in the Sudan–Sahel and Sudan climatic areas. The sub-humid Sudan–Sahel zone is characterized by annual rainfall of ~600–900 mm, concentrated in a short rainy season (June–September), high temperatures, and a prolonged dry season, resulting in open savanna vegetation and high inter annual rainfall variability. The humid Sudanian zone, receiving ~900–1100 mm of rainfall annually, experiences a longer and more reliable rainy season lasting 5–6 months, slightly lower mean temperatures, and denser savanna to woodland vegetation. Overall, the north–south rainfall gradient governs the length of the growing season, water availability, and agricultural potential across these climatic zones [32]. The Goué site featured flat terrain, fertile loamy–sandy soils, and consistent water availability from a local dam. The site mean annual temperature ranged between 27 and 28°C. The Léguéma site presents with slightly uneven terrain, clay–sandy soils and is supplied by surface water sources such as seasonal streams and small rivers. Mean annual temperature ranged between 26 to27°C. Sampling was performed following a 500 × 1000 m grid with meshes of 250 × 250 m (Fig. 1). In a radius of 20 meters of each point of the grid, leaves from up to ten individuals were collected for the main plant species. Importantly, leaf samples were collected regardless of the presence of visible disease symptoms. A total of 1068 samples, collectively representing 25 plant species, were collected with 495 samples from Goué and 573 from Léguéma. All samples were conserved in envelopes and dried using an oven at 50°C overnight and stored at room temperature before molecular analysis [30].

### Deoxyribonucleic acid extraction

Plant total DNA was extracted from 20 mg of leaves for all samples using the DNeasy Plant Mini Kit (Qiagen) according to the manufacturer’s instructions. DNA extracts were stored at −30°C before use.

### Sample barcoding for plant identification

Sequencing of the *matk* and *rbcl* genes were performed on samples for which no confident genus or species visual identification was obtained. PCR were conducted with the primers and conditions described in [33] before direct Sanger sequencing (Macrogen, South Korea). After quality control, sequences were classified using the RDP classifier [34] against a database of *matk* and *rbcl* plant sequences obtained from GenBank in August 2017.

### Sample amplification for Illumina sequencing

The 1068 samples were processed using the RCA-RA-SGS sequencing procedure described in [30]. Briefly, after a Rolling Circle Amplification (RCA; [35]) using the Templiphi kit (Illustra GE Healthcare), a random amplification (RA) was performed to tag individual samples. Samples were treated in duplicate. Equimolar pools of amplicons were then sent to Azenta (USA) for short-read sequencing. Libraries were constructed after a Covaris shearing of fragment amplicons to the size range suitable for Illumina sequencing and 2X150 bp paired-ends sequencing was performed on the Illumina HiSeq 2500 and HiSeq X Ten platforms.

### Short-read sequence data analysis

After a first rapid classification against a custom database using Kraken2 [36], raw sequence fragments (hereafter referred to as reads) obtained by Illumina sequencing were classified following the pipeline described in [30]. Briefly, after quality control, reads were demultiplexed and pairs were merged when possible. After dereplication, an initial fast taxonomic assignment of dereplicated sequences was performed using the BLASTn and BLASTx equivalent algorithm of DIAMOND v2 [37] and VSEARCH v2.21 [38], respectively, on a database restricted to the geminiviruses and their satellites and obtained from GenBank on October 2017. Reads presenting with significant similarities from the database were then clustered and more precisely classified onto the begomovirus, mastrevirus and satellites databases using pplacer [39] and the BoSSA R package (https://cran.r-project.org/package=BoSSA).

### Long-read sequencing and data analyses

To obtain full virus sequences, RCA-MinION sequencing [31] was performed on samples detected as positive using the RCA-RA-SGS procedure. Flongles FLO-FLG001 (Oxford Nanopore Technologies) were used for sequencing. Conveniently, for each Flongle, amplicons from ten samples were multiplexed along with a positive and a negative control.

After accurate basecalling using Guppy v4.015 (https://nanoporetech.com/) and demultiplexing and adapter removal using Porechop v0.2.4 (https://github.com/rrwick/Porechop), full CRESS DNA genomic components were assembled using two complementary pipelines as described in [40]. Sequence coverage statistics of assembled genomic components were estimated after mapping the raw reads back to the assembled CRESS DNA sequences using minimap2 [41].

### Cloning and sequencing of complete circular replication-associated protein encoding single stranded deoxyribonucleic acid genome components

Concomitantly, a fraction of the positive samples, as assessed using the RCA-RA-SGS procedure, were subjected to rolling circle amplification and restriction fragment length polymorphism analysis (RCA-RFLP), cloning and Sanger sequencing as described elsewhere [15]. Resulting sequences were edited and assembled using Geneious R8 software (Biomatters Ltd).

### Phylogenetic and recombination analysis of assembled sequences

Sequences obtained using either RCA-MinION or RCA-RFLP were subjected to a BLASTn search for preliminary species assignment. Sequence alignments were then constructed for each identified virus genus. Along with the sequence obtained from this study, the alignments included representative sequences that were isolated from samples collected in Africa (as obtained from GenBank in June 2022). Alignments were performed using MAFFT v7.490 [42]. Pairwise identity comparisons of nucleotide sequences were performed using the “ape” R package [43]. Based on ICTV endorsed genus-specific sequence identity-based species demarcation criteria (91% over the full length of the DNA-A or DNA-A-like component of begomoviruses; [44]; 78% for mastreviruses; [45]; 88% for alphasatellites; [46]; and 91% for the tolecusatellites; [9], sequences were assigned to species.

Maximum likelihood phylogenetic trees were inferred using FastTree 2 [47] with the “gtr” and “gamma” parameters. Recombination events were then detected within alignments using the RDP [48], GENECONV [49], BOOTSCAN [50], MAXCHI [51], CHIMERA [52], SISCAN [53], and 3SEQ [54] methods implemented in the RDP5 program [55]. As we were primarily interested in uncovering events that have plausibly occurred within the vicinity of the sampled area, a “group” analysis was used in RDP5 so as to detect only recombination events involving sequences closely related to those detected in Burkina Faso. All detected recombination events were then manually inspected and filtered (both parental sequence identified and minimum % of identity between parent and recombinant of 95% over recombinant tracks) to identify those recombination events that were likely to have occurred in the vicinity of sampling sites in the fairly recent past. Besides the grouping scheme, default RDP5 settings were used and recombination events were considered significant when detected by at least four different recombination detection methods. Additionally, inter-component recombination (e.g. between DNA-A like and DNA-B sequences or between DNA-A like and alphasatellite sequences) were inspected using all-versus-all BLASTn searches. BLAST results were filtered (E-Value <0.001; % identity ≥ to 95%) to restrict the result sets to only very similar hits. Similarities between components were later confirmed after manual inspection of the putative events using standard phylogenetic analyses.

### Virus regulatory element analysis

Putative iteron sequences (i.e. repeated sequence motifs associated with Rep protein binding during initiation of replication; [56] were searched within sequences obtained from the RCA-MinION and RCA-RFLP using custom scripts. Briefly, repeated short sequences (size ranging from to 7 to 11 nt) found at least three times within the CR (with a maximum edit distance of 1 from one another) were selected. Search was performed on the viral and complementary strand of the DNA. Up to three putative iteron sets (those with the largest number of repeat and repeat size and the less mutations) were considered for further analysis. Iteron sequences were then compared between all sequences to construct an iteron compatibility matrix. A pair of sequence was dubbed compatible when they present a minimum of one common iteron repeat.

### Structure of plant–virus association and host range measurements

Contingency matrices of numbers of per plant species positive samples to each viral species were used for community structure analysis. Bipartite networks were constructed and visualized using the bipartite [57] and igraph R packages [58]. The weighted modularity measurements were performed using the DIRTLPAwb+ algorithm [59]. The significance was assessed by comparing the observed modularity score to those obtained from 1000 randomized networks generated using the nullmodel function from the vegan R package [60] with the r2dtable method, which preserves the row and column sums of the original interaction matrix.

## Results and discussion

### Plant diversity within agro-ecosystems from Goué and Léguéma

To assess the distribution of CRESS DNA viruses across agro-ecosystems, 1068 plant samples were collected from the Goué (*n =* 495) and Léguéma (*n =* 573) localities following a grid sampling strategy. These samples collectively represented 25 species from 17 identified genus (Table 1). The majority of samples were of uncultivated plant species (81%, 866/1068) and species presenting with an annual life cycle (70%, 739/1068, 13 species). The 30% of plants that were not classified annuals were classified as either perennial (14%, 149/1068, four species) or annual/perennial (annual or perennial depending on environmental conditions, 16%, 168/1068, eight species) (Table 1). Fourteen out of the 25 identified plant species were common to both sites, indicating a substantial floristic overlap, whereas five and six species (representing five and six plant genera) were specific to Goué and Léguéma, respectively (Table 1). Indeed, spontaneous herbs, mainly from Amaranthaceae, Asteraceae, and Euphorbiaceae, showed variable distributions between sites, indicating that microclimate, soil, and local management strongly influence plant communities [61–63].

**Table 1:** Summary of sampled plant species from Goué and Léguéma in Burkina Faso.

Crop status	Family	Species	Seasonality	Goué	Léguéma
Cultivated	Malvaceae	*A. esculentus*	Annual	30	30
	Malvaceae	*Hibiscus* sp.	Annual/Perennial	54	-
	Solanaceae	*C. annuum*	Perennial	10	-
	Solanaceae	*C. frutescens*	Perennial	31	2
	Solanaceae	*S. lycopersicum*	Annual	-	45
Uncultivated	Amaranthaceae	*Amaranthus hybridus*	Annual	-	1
	Amaranthaceae	*Amaranthus spinosus*	Annual	22	52
	Amaranthaceae	*Amaranthus viridis*	Annual	66	63
	Asteraceae	*Ageratum conyzoides*	Annual/Perennial	9	62
	Boraginaceae	*Heliotropium indicum*	Annual/Perennial	10	-
	Convolvulaceae	*I. alba*	Annual	9	30
	Euphorbiaceae	*Euphorbia heterophylla*	Annual	-	44
	Euphorbiaceae	*Euphorbia hirta*	Annual	50	64
	Hydroleaceae	*Hydrolea* sp.	Annual/Perennial	-	4
	Lamiaceae	*Leucas martinicens*	Annual	10	18
	Malvaceae	*Melochia* sp.	Annual/Perennial	9	2
	Malvaceae	*S. acuta*	Perennial	25	68
	Malvaceae	*Sida rhombifolia*	Annual/Perennial	-	7
	Malvaceae	*Sida* sp.	Perennial	13	-
	Nyctaginaceae	*Boerhavia erecta*	Annual	70	49
	Pedaliaceae	*Sesamum* sp.	Annual	-	3
	Rubiaceae	*Mitracarpus* sp.	Annual/Perennial	10	-
	Rubiaceae	*Mitracarpus villosus*	Annual	40	5
	Solanaceae	*Physalis ixocarpa*	Annual	17	21
	Undetermined	Undetermined	Undetermined	10	3

### Cultivated plants and perennials harbor high virus prevalence

All samples were first processed using the RCA-RA-Illumina procedure [30] in order to characterize the diversity of CRESS DNA viruses circulating at the sites. Global similarity search analysis resulted in 20% of the ~100 million raw reads being assigned to CRESS DNA viruses (see Supplementary Fig. S1a).

**Figure 2:**
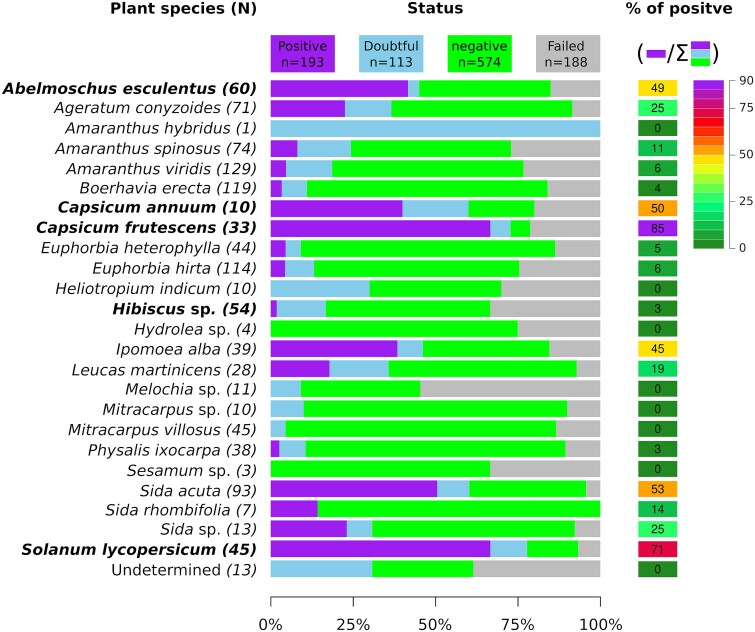
Proportions of plant species for each assignment status and infection rates for each plant species. Plant species names in bold correspond to cultivated plants. The numbers in brackets refer to numbers of collected samples. Histogram colors refer to the infectious status such as positive (in purple), doubtful (in light blue), negative (in green), and failed (in gray). The number of infected samples for each plant species is indicated in the cell on the right. Cells are colored according to the infection rate (see the scale on the top right).

These reads were obtained from 193 of the 1068 samples (102/495 for Goué and 91/573 for Léguéma; Fig. 2) making it a global CRESS DNA virus positivity rate of 18%. These plants were from eight families, twelve genera and 17 species. Of the 193 positive plants, 82 were cultivated and 111 were uncultivated.

The 82 positive cultivated plants belonged to two families (*Malvaceae* and *Solanaceae*) comprising four genera (*Abelmoschus, Hibiscus, Capsicum*, and *Solanum*) and five species (Supplementary Table S1), while the 111 positive uncultivated plants belonged to eight families (*Amaranthaceae, Asteraceae, Convolvulaceae, Euphorbiaceae, Lamiaceae, Malvaceae, Nyctaginaceae, Solanaceae*) comprising eight genera (*Amaranthus, Ageratum, Ipomoea, Euphorbia, Leucas, Sida, Boerhavia, Physalis*) and twelve species (Supplementary Table S1). The cultivated plants *Abelmoschus esculentus* (49%), *Capsicum frutescens* (85%), *Capsicum annuum* (50%), and *Solanum lycopersicum* (71%) and the uncultivated plants *Ipomoea alba* (45%) and *Sida acuta* (53%) presented with the highest infection rates (Fig. 2).

Significantly higher positive rates were noted for cultivated plants species than uncultivated species (Chi-squared test, *P*-value <.001) with averages of 41% (82/202) and 13% (111/866), respectively. Other comparisons showed that perennial plants (Goué: 62%, 49/79; Léguéma: 39%, 27/70) were significantly more infected than both annual plants (Goué: 16%, 49/314; Léguéma: 12%, 50/425) and perennial/annual plants (Goué: 4%, 4/92; Léguéma: 19%, 14/75) in both Goué and Léguéma (all four Chi-squared tests yielding *P*-values <.001).

### High diversity of geminiviruses and associated satellites across the agro-ecosystems

The 193 positive samples were then processed for full genome sequencing and sequences were successfully obtained for 106 samples (total of 222 viral contigs; Supplementary Fig. S1a and b; Supplementary Table S2). A total of 127 contigs were assigned to members of the *Geminiviridae* family (Supplementary Fig. S2), 43 to members of the *Alphasatellitidae* family (Supplementary Fig. S3a) and 52 to members of the *Tolecusatellitidae* family (Supplementary Fig. S3b). Geminivirus contigs included 109 complete sequences while alphasatellite and betasatellite contigs included 38 and 52 complete sequences, respectively. Among the assembled sequences likely to be of geminivirus origin, 94% (119/127) were most closely related to members of the *Begomovirus* genus (with 93 DNA-A/DNA-A like and 26 DNA-B components) and the remaining 6% (8/127) to members of the *Mastrevirus* genus*.*

Plant virology in general has benefited from the extension of viral metagenomic surveys beyond cropping systems where it has enabled the study of plant viral diversity and evolution at the scale of entire ecosystems [28, 64, 65]. Accordingly, in our study, a large amount of viral diversity was uncovered (Table 2). Besides one *Mastrevirus* species (chickpea chlorotic dwarf virus, CpCDV), identified on four plant species, these geminiviruses included 16 *Begomovirus* species, among which seven species have been already described and nine should be assigned to new species. Names were proposed (see Table 2 for details) based on the presence or absence of symptoms. DNA-B sequences from two different *Begomovirus* species were also identified during the surveys, one of which have already been described from Burkina Faso [66]. The second species was characterized for the first time and was genetically distant from DNA-B described elsewhere. It presents similarities of 61% and 73% with desmodium mottle (KY294726) and cleome leaf crumple (JF694464) begomoviruses from the Old and New World, respectively. For the alphasatellites, of the seven species identified in our study, three were already described, whereas the other four should each represent new species. The six tolecusatellite species were all members of the *Betasatellite* genus. Two species were already described and four were probable new species.

**Table 2:** List of the virus species, including putative new species, described in this study.

Family	Genus	Virus common names	Virus species	Putative new species	Number of samples
*Alphasatellitidae*	*Colecusatellite*	Cotton leaf curl Gezira alphasatellite	*Colecusatellite gossypigeziraense*	No	13
		Cotton leaf curl Gezira alphasatellite 2	*Colecusatellite gossypigeziraensecundi*	Yes	2
		*S. acuta* yellow leaf curl alphasatellite	*Colecusatellite sidacutae*	Yes	14
	*Gosmusatellite*	Cotton leaf curl Gezira alphasatellite 3	*Gosmusatellite gossypicameroonense*	No	2
		Okra vein thickening alphasatellite	*Gosmusatellite abelmosentus*	Yes	1
		Okra yellow crinkle Cameroon alphasatellite	*Gosmusatellite abelmoschuscameroonense*	No	8
	Unclassified	Cotton leaf curl Gezira alphasatellite 1	Unclassified	Yes	3
*Geminiviridae*	*Begomovirus*	*Boerhavia erecta* associated virus 1	*Begomovirus boerhaviae*	Yes	2
		*B. erecta* associated virus 2	*Begomovirus boerectae*	Yes	2
		Cotton leaf curl Gezira virus	*Begomovirus gossypigeziraense*	No	12
		Ipomoea mild leaf curl virus	*Begomovirus ipomoalbae*	Yes	5
		Ipomoea yellow crinkle virus	*Begomovirus ipomogouense*	Yes	6
		Okra vein thickening virus	*Begomovirus abelmosentus*	Yes	1
		Pepper yellow vein Mali virus	*Begomovirus capsicummaliense*	No	19
		Pepper yellow vein Mali virus DNA-B	*Begomovirus capsicummaliense*	No	25
		*S. acuta* yellow leaf curl virus	*Begomovirus sidacutae*	Yes	6
		Sida yellow mosaic Burkina Faso virus	*Begomovirus sidafasoense*	Yes	2
		Sida yellow mosaic Burkina Faso virus DNA-B	*Begomovirus sidafasoense*	Yes	1
		Sweet potato leaf curl virus	*Begomovirus ipomoeae*	No	3
		Tomato leaf curl Cameroon virus	*Begomovirus solanumghanaense*	No	1
		Tomato leaf curl Ghana virus	*Begomovirus solanumghanaense*	No	12
		Tomato leaf curl Mali virus	*Begomovirus solanummaliense*	No	21
		Tomato vein thickening virus	*Begomovirus tomatosicum*	Yes	1
	*Mastrevirus*	Chickpea chlorotic dwarf virus	*Mastrevirus cicerparvi*	No	8
*Tolecusatellitidae*	*Betasatellite*	*B. erecta* associated betasatellite	*Betasatellite boerhaviae*	Yes	5
		Cotton leaf curl Burkina Faso betasatellite	*Betasatellite gossypiburkinafasoense*	No	22
		Cotton leaf curl Gezira betasatellite	*Betasatellite gossypigeziraense*	No	4
		*S. acuta* associated betasatellite	*Betasatellite sidacutae*	Yes	5
		Tomato leaf curl Ghana betasatellite 3	*Betasatellite solanighanaensetertii*	Yes	9
		Tomato leaf curl Ghana betasatellite 4	*Betasatellite solanighanaensequarti*	Yes	7

**Figure 3:**
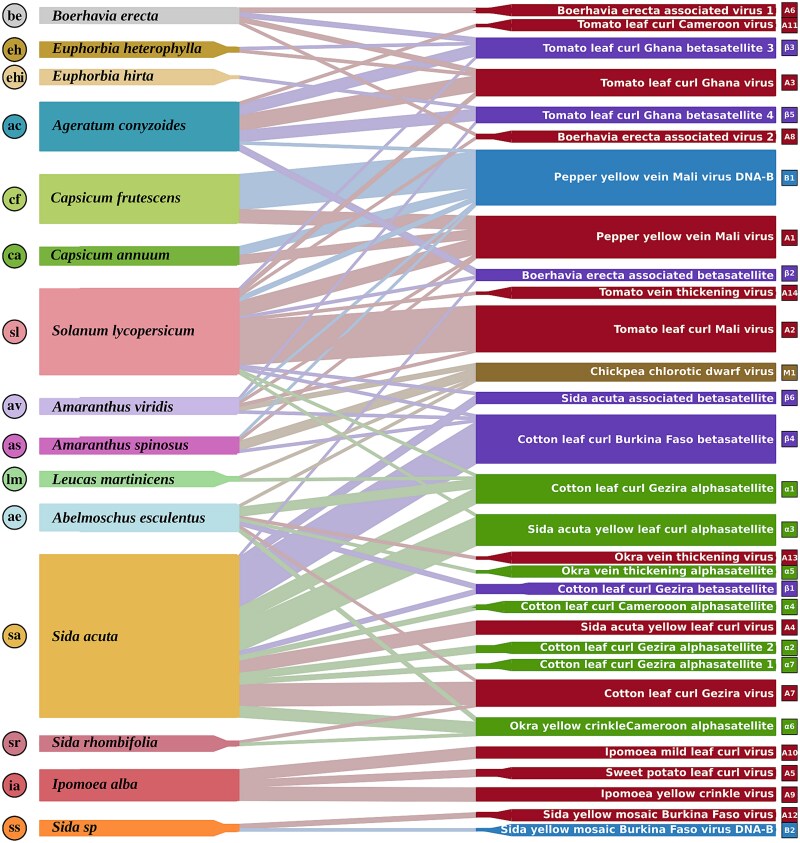
Interaction graph representing the association between plant species (left side of the diagram) and viral species (right side of the diagram) with the size of the boxes and links proportional to the number of positive plant samples. Links are colored according to the viral species type with begomovirus DNA-A in red, begomovirus DNA-B in blue, *Mastrevirus* in dark yellow, alphasatellite in green and betasatellite in purple.

Overall begomoviruses DNA-A sequences were found in a total of 13 plant species from nine genera and seven families (from one to five plants per DNA-A lineage with a mean of 1.8, Fig. 3). The DNA-B sequences were associated with seven plant species from five genera and four families (from one to six hosts per DNA-B lineage with a mean of 3.5). It is noteworthy that there was a clear association of the pepper yellow vein Mali virus (PepYVMLV) DNA-A and PepYVMLV DNA-B components [15, 66] across a range of different host species including *Amaranthus. viridis, Amaranthus. spinosus, C. annuum, C. frutescens* and *S. lycopersicum*, confirming that co-infection with both components is likely important for efficient infection and transmission of PepYVMLV [66].

The seven alphasatellite species were almost exclusively detected associated with *Malvaceae* plants belonging to three different species (*A. esculentus, S. acuta, Sida rhombifolia*; Fig. 3). The exceptions were cotton leaf curl Gezira alphasatellite (CoLCGA) and *S. acuta* yellow leaf curl alphasatellite (SaYLCA) that, besides Malvaceae plants, were also detected in *Leucas martinicensis* and *S. lycopersicum*, respectively. Conversely, betasatellites from the *Tolecusatellitidae* family were detected in nine hosts representing seven genera and six families (from two to four, mean of 3.0).

### Host–virus association networks display high modularity

**Figure 4:**
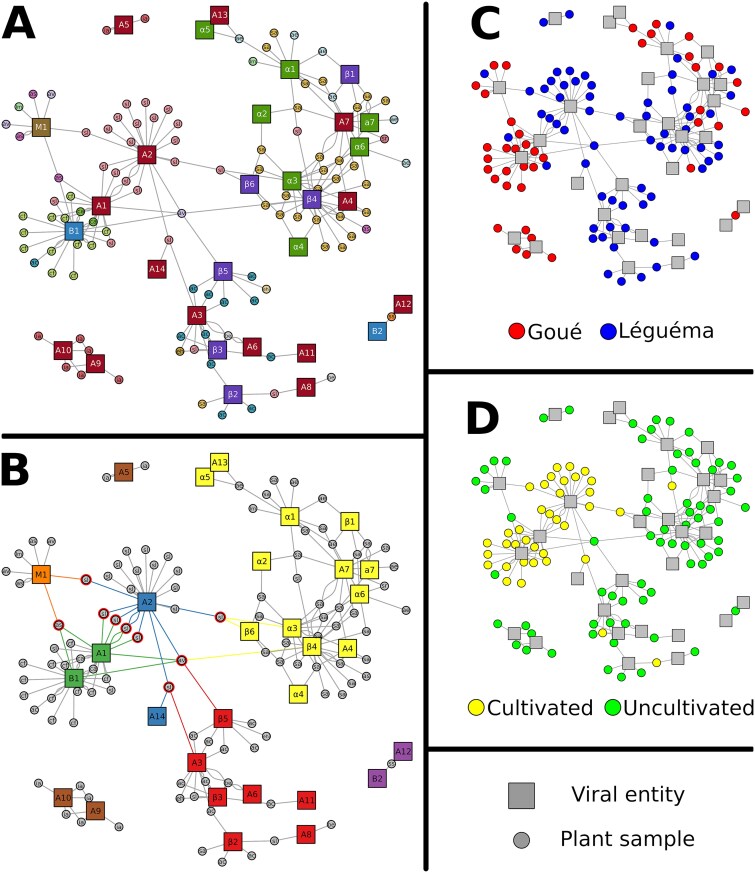
Bipartite graphs representing the community structure of geminiviruses from two localities of Burkina Faso. Square nodes represent virus species. Circular nodes represent plant samples. Graphs are colored according to (a) viral species and plant species following colors used in Fig. 3; (b) modules of infection with samples infected with viruses from different modules indicated with red outer circles; (c) sampling site with Léguéma in red and Goué in blue; and (d) cultivated or uncultivated status. Letters A, B, M, α, and β indicate cluster numbers for begomovirus DNA-A/A-like, begomovirus DNA-B, mastrevirus, alphasatellites and tolecusatellites, respectively. Codifications are presented in Fig. 3.

Having sampled a large number of individuals from several species, we were able to determine the effective host ranges of geminivirus and their associated satellites under natural conditions. These associations were represented within an infection network linking viral species to plant samples. In the network, two classes of nodes were represented: viral species nodes (squares in Fig. 4), and host plant sample nodes (circles in Fig. 4). Each plant sample node is linked with all the nodes of all virus species that were detected in that plant sample. There is no direct link between any two plant sample nodes and between any two virus species nodes.

Globally, the network was composed of four connected components (groups of nodes connected together but without connection to another group) with one large component gathering 83% of the viral species (25/30) and 92% of the plant samples (98/107) (Fig. 4; Supplementary Fig. S4). Importantly, all but two plant species (*I. alba* and *Sida* sp.) were present within this large component. These two were the sole species associated with the three other smaller components: Two components were composed of viruses exclusively infecting *I. alba* (eight samples) and one component was composed of a single unidentified plant from the *Sida* genus that was co-infected with a begomovirus DNA-A and DNA-B component.

Modularity, a measure of the strength of the division of a network into groups of viruses and hosts that preferentially interact with one-another, was significant (*P*-value <10^−3^) with five and six modules detected for Goué and Léguéma localities, respectively, and maximized with seven modules (Fig. 4a and b; Supplementary Fig. S4, and Supplementary Table S3). Five modules were composed of *Begomovirus* species without satellites (module #1 in blue; module #2 in green; module #4 in purple; module #5 in brown and module #6 in orange; Fig. 4b and Supplementary Fig. S4). All the satellites, along with other virus species, were gathered in two modules (module #3 in red and module #7 in yellow; Fig. 4b and Supplementary Fig. S4). It is interesting to notice that the modules containing cultivated plants also contained uncultivated plants (Fig. 4d and Supplementary Fig. S4). In particular, within the largest module (module #7), uncultivated malvaceous plants from the *Sida* genus were associated to viruses that otherwise infect the cultivated species *A. esculentus* (okra). In other modules (modules #3 and #6), uncultivated plants from the *Ageratum* and *Amaranthus* genera were associated with viruses that also infect cultivated solanaceous plants (tomato). These uncultivated species could therefore play an essential role as reservoirs of crop-infecting viruses, sustaining these viruses within agro-ecosystems during inter-cropping periods [23].

It must be emphasized here that several satellites molecules were detected in samples, without the detection of any helper viruses, potentially indicative of false negative results. Indeed, of all the samples that were positive in our survey, 32% presented with satellites or DNA-B component in the absence of begomovirus DNA-A or DNA-A like components. The absence of detected helper viruses may be associated with amplification bias associated to the RCA [67] resulting in the enrichment of satellites which might impede the detection of larger components. We thus tested how the modularity results was robust to a potential detection bias and again modularity was found highly significant (*P*-value <10^−3^) when considering the interaction network limited to the samples for which a putative helper virus was detected (Supplementary Fig. S5).

### Inter-module co-infection might be restricted

Frequent co-infections were also observed, with 61 of the 106 plants that were positive for the presence of any CRESS DNA elements, containing two or more viral species. Of these 61 samples of “mixed infection”, 51 presented with co-infections with components of multiple types. The most frequent combinations of inter-component co-infections were DNA-A/betasatellites (*N =* 22), DNA-A/DNA-B components (*N =* 13), DNA-A/alphasatellites (*N =* 12) and alphasatellites/betasatellites (*N =* 18). This tendency was more pronounced within the largest module (highlighted in yellow) where 25/34 of the plant samples were infected by viruses and at least one alphasatellite, and 23/34 of the samples were infected by viruses and at least one betasatellite. Sixteen out of 34 were infected by species of both satellite types. It confirmed that the frequency of satellites in the field is high and that several satellites can be found associated with a single helper virus [68, 69].

**Figure 5:**
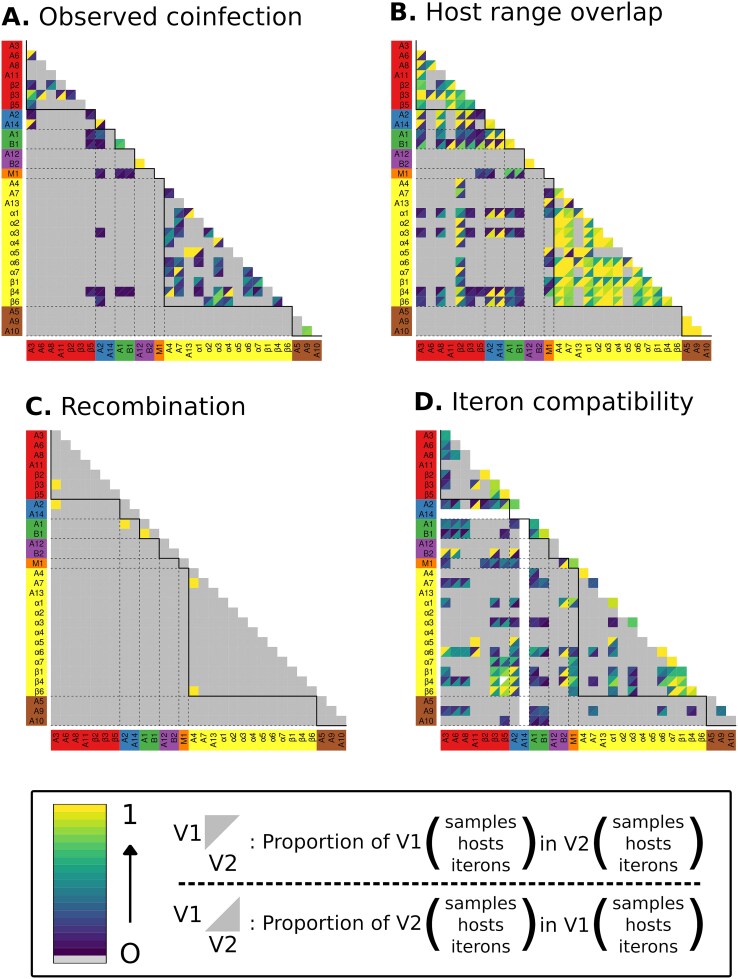
Hemi-matrices representing the co-infection frequencies (a), the host-range overlap (b), the recombination events (c) and the putative iteron compatibility (b) between viruses from all the identified species. Plain and dashed lines represent the clusters of infection delimitation. Clusters affiliations are color-coded, over matrix labels. Hemi-matrix A, B, and D values ranged from zero to one, according to the color scale shown on the bottom panel. Panel B for recombination are binary (0 or 1), where 1 indicates the detection of recombination between the viral entities. When appropriate, cells are divided into two areas with the top left area and the bottom right area colored according to the statistics of the species in rows relative to the species in column and of the species in column relative to the species in row, respectively. White cells represent missing data.

Whereas the networks from Goué and Léguéma gathered 41 and 65 samples, respectively, a single sample (one *A. spinosus* sample) from Goué and eight from Léguéma (seven *S. lycopersicum* and one *A. viridis* samples) presented with co-infection of viruses belonging to multiple modules (Figs 4B and 5A). Knowing the host range of each virus, it was possible to randomize infection patterns and compare the observed number of co-infection involving viruses from different modules to the permutations. As for Goué, both permutation and observation involve at most a single inter-module co-infection, our test was inconclusive. However, for Léguéma, the test yield a *P*-value of 7.13 × 10^−2^ that samples presented with fewer inter-modules co-infections than expected by chance given the known virus host ranges (Fig. 5b). Although it would requires confirmation, this marginally significant *P*-value for Léguéma may indicate that interactions between viruses and hosts across different modules are not solely determined by host-range considerations. It is indeed also possible that exclusion or synergistic processes contribute, at least in part, to the structuring of virus associations within modules [26].

### Deoxyribonucleic acid exchange by recombination indicates that viruses from different modules sometimes interact

Recombination is considered a central driver of the molecular evolution of CRESS DNA viruses [70], and has been implicated in the emergence of multiple viruses with altered phenotypes [4, 71, 72]. To determine if this evolutionary process was important within the CRESS DNA community characterized here, per component type alignments were constructed and analyzed for the presence of recombination. Of the 98 intra-component events that were detected, based on percentage of identity filter, three may have occurred within the vicinity of the sampled area and in a relatively recent past (Table 3, Fig. 5c). All the events involved begomovirus DNA-A component sequences. The first involved a single isolate from the ToVTV species with recombinant parents from the ToLCMLV and ToLCGHV species. The portion of sequence in this recombinant that was obtained from ToLCGHV spanned genome positions including the full CP, C3 and C2 ORFs along with a fraction of the C1 ORF. Whereas the parental sequences were not from the same infection module (i.e. modules are not fully isolated groups of plant and viruses but are rather composed of groups of plant and viruses that preferentially, but no exclusively, interact with one another), they were found in co-infection within the same *S. esculentum* plant from which this recombinant was also isolated.

**Table 3:** Intra and inter-component recombination.

Type	Recombinant^a^	Parent(s)	Position in recombinant sequence^b^	Genomic feature	*P*-value^c^
DNA-A/DNA-A	ToVTV	ToLCMLV–ToLCGHV	528–1990	Full V1, C2, and C3 ORFs; partial C1 ORF	5.50e-143
DNA-A/DNA-A	ToLCMLV	ToLCMLV–PepYVMLV	1120–2569	Full C2 ORF, partials C1, and C3 ORFs	3.12e-63
DNA-A/DNA-A	SaYLCV	SaYLCV–CLCuGeV	279–1042	Partial V1 and V2 ORFs	3.93e-29
DNA-A/DNA-B	PepYVMLV DNA-B	PepYVMLV DNA-A	2502–2685 [2627–2811]	Non-coding	2.82e-15
DNA-A/betasatellite	ToLCGHB3	ToLCGHV	1054–1116 [2754–2692]	Non-coding	2.18e-26
DNA-A/betasatellite	SaAB	SaYLCV	727–763 [1128–1164]	Non-coding	6.32e-12

^a^Note that for inter-component recombination, it is uncertain which of the sequence pair is the donor (parental sequence) and the receiver (recombinant).

^b^For inter-component recombination, position in the parental sequence is indicated within brackets.

^c^Best *P*-values among the sequence pair.

CLCuGeV: Cotton Leaf Curl Gezira Virus.

PepYVMLV: Pepper yellow vein Mali virus.

ToLCGHB3: Tomato leaf curl Ghana betasatellite 3.

ToLCGHV: Tomato leaf curl Ghana virus.

ToLCMLV: Tomato leaf curl Mali virus.

ToVTV: Tomato vein thickening virus.

SaAB: *S. acuta* associated betasatellite.

SaYLCV: *S. acuta* yellow leaf curl virus.

The second event was detected within one of the 19 sequences belonging to the ToLCMLV species, with parents from the ToLCMLV and PepYVMLV species. Again, the parental sequences were not from the same infection module but were both found in co-infections in *A. viridis* and *S. lycopersicum*. The *S. lycopersicum* plant within which the recombinant sequence was found was also co-infected by viruses from both parental species. The portion of this recombinant genome derived from PepYVMLV included the full C2 ORF and fractions of the C1 and C3 ORFs.

The third recombination event was detected within two of the six sequences from the SaYLCV species, involving parents from the CoLCuGeV and SaYLCV species. The parental sequences were from the same infection module. The two recombinant sequences were isolated from *S. acuta* plants and one of these was found in co-infection with both parental species. The genome regions of these recombinants that was derived from CoLCuGeV included a fraction of V2 ORF and almost the entirety of the V1 ORF.

Inter-component recombination events were detected using sequence similarity searches. As for intra-component recombination, only events with recombination tracks presenting with high identities (>95%) were considered. Three events were uncovered (Table 3, Fig. 5c) with one between begomovirus DNA-A and DNA-B components and two between begomovirus DNA-A components and betasatellites. The DNA-A/DNA-B events involved a probable partial rewrite of the common region of the PepYVMLV DNA-B with that of PepYVMLV DNA-A. Both species were forming the module #2 Fig. 4b and Supplementary Fig. S4). All but two of the PepYVMLV DNA-B sequences displayed high similarities with PepYVMLV DNA-A over a ~ 185 base stretch in the intergenic region and upstream of the begomovirus origin of replication. Interestingly, this section also contained some of the putative iteron repeats, i.e. repeated sequence motifs associated with Rep protein binding during the initiation of virion strand replication [56], possibly improving the compatibility of these DNA-B components (Fig. 5d) for trans-replication by the PepYVMLV DNA-A encoded Rep protein [66].

The first of the DNA-A/betasatellite events involved viruses from the ToLCGHV and ToLCGHB3 species with the non-coding region of the latter presenting similarities with a fraction of the IR of the former. Both viruses belonged to the same infection modules and were found in co-infection in six of the seven plants where ToLCGHB3 was found. The second event involved viruses from SaYLCV and SaAB. A similarity was found between a fraction of the IR of both virus species. However, no co-infections were observed between these viruses, despite them being from the same infection modules and having largely overlapping host ranges.

### Community structure, satellite molecules, and the evolutionary potential of plant-infecting geminiviruses and associated satellites

The targeted metagenomic approach employed in this study proved highly effective for resolving the structure of plant virus communities at the scale of an entire agroecosystem. By capturing both virus diversity and host associations, this approach represent an important step toward understanding the processes via which some viruses emerge as local or global threats to crop production [73–75]. In agreement with previous surveys, we confirmed the high prevalence and diversity of CRESS DNA viruses in agricultural environments [40, 76–78]. Going further, we demonstrate that this diversity was not randomly distributed, but instead organized into coherent infection modules comprising viruses that appear co-adapted to particular host groups. It should first be noted that the present study did not specifically investigate the role of insect vectors in shaping virus community structure. Nevertheless, vector ecology (i.e. *B. tabaci* for begomovirus transmission) likely contributes substantially to the distribution of viruses and to the organization of the observed modules. Factors such as vector host preference, feeding behavior, dispersal, and seasonal fluctuations in population size may strongly influence which plant species become connected through virus transmission within the agroecosystem. As a result, the observed modular structure may not only reflect host compatibility or virus adaptation to particular plant taxa, but also the ecological behavior and the dynamics of insect vectors of viruses in the agroecosystems. Future research that takes into account the feeding preferences of insect vectors, their seasonal patterns, their migratory capacity and their transmission dynamics will undoubtedly help to clarify the processes that shape the structure and dynamics of these viral communities.

Despite the strong modularity observed, modules were not confined to either cultivated or uncultivated plants. Instead, all modules containing cultivated hosts also included uncultivated plant species, highlighting the ecological continuity between crop and non-crop compartments. The largest module, in terms of both plant sample number and virus species richness, was dominated by plants from the *Sida* genus, which were among the most frequently sampled perennial plants. Importantly, viruses detected in *Sida* plants were also found infecting the cultivated crop *A. esculentus*, indicating extensive host sharing between perennial weeds and crops and highlighting again the importance of weeds in plant virus disease [79]. Other modules further illustrated this pattern: uncultivated plants belonging to the genera *Ageratum* and *Amaranthus* (highlighted in red and orange, respectively) were associated with viruses also detected in cultivated solanaceous plants. These findings support the idea that uncultivated species play a key role as reservoirs of crop-infecting viruses, allowing viral populations to persist locally during inter-cropping periods and recolonize crops when susceptible hosts become available. This reservoir function has been repeatedly emphasized in theoretical and empirical studies of plant virus epidemiology [23, 80–82], and our results provide community-scale evidence for its importance in CRESS DNA virus systems.

Notably, satellite molecules were found to be predominantly associated with non-cultivated plants, that represent important ecological niches for sustaining the full complexity of CRESS DNA virus–satellite interactions. Going further, the potential role of these satellites in fostering the successful infection of crop specific viruses in non-crop host would need to be clarified: Experimental and field-based studies have shown that certain satellites can modulate symptom expression, viral accumulation, and host range of their helper viruses, in some cases enabling infection or persistence in hosts that are otherwise poorly permissive [83–85]. If satellites from the uncovered communities had similar role, it would promote the maintenance of crop-infecting viruses in non-crop reservoirs and increase opportunities for subsequent spill-back into cultivated hosts [80].

Finally, the exchange of DNA between viruses in different modules through recombination may suggest that, from an evolutionary perspective, viruses could potentially interact across module boundaries, even though ecological associations appear strongly partitioned. The permeability of module boundaries might be mediated by a limited number of shared or “bridge” hosts that could connect otherwise distinct virus–host modules. The identity and importance of such bridge hosts may vary seasonally as cropping patterns and host availability change. Nevertheless, they would likely include the so called “mixing vessels” species [23], defined here as species found infected with viruses belonging to multiple modules, such as *A. viridis* and *S. lycopersicum* which were associated with viruses from five and four modules, respectively. In addition to acting as year-round reservoirs for CRESS DNA viruses, shared hosts may be particularly important with respect to fostering mixed infections of, and recombination between, viruses in these different modules, especially during the dry and post-harvest seasons [65, 86, 87].

Together, these results emphasize that uncultivated plants are not merely passive components of agroecosystems, but most probably active contributors to virus persistence, diversification, and emergence. Understanding further how viral community structure, host connectivity, insect vector feeding preferences and population dynamics, together with seasonal fluctuations interact will be essential for predicting and managing future risks to crop production.

## Supplementary Material

Supplementary_Tables_20260526_ycag196

## Data Availability

All sequences are available on NCBI GenBank under the accession numbers detailed in Supplementary Table S2.
