# Supplementary material for: Geminivirus diversity and community structure at the scale of agro-ecological systems in two localities of Burkina Faso
Source: ISME Commun. 2026 Jul 8;6(1):ycag196. doi: 10.1093/ismeco/ycag196 (PMC13452460; doi:10.1093/ismeco/ycag196)
Supplement: Supplementary file 1 [file supplementary_tables_20260526_ycag196.docx]

**Supplementary Table 1: Percentage of infected cultivated and uncultivated plants collected from Goué and Léguéma in Burkina Faso and full-genome sequences statistics**

| **Locality** | **Host status** | **Host family** | **Host genus** | **Host species** | **RCA-RA-Illumina^$^** | **RCA-RA-MinION^£^** | **RCA-RFLP^£^** |
| --- | --- | --- | --- | --- | --- | --- | --- |
| Goué | Cultivated | *Malvaceae* | *Abelmoschus* | *Abelmoschus esculentus* | 96% (23/24) | 22% (5/23) | 13% (3/23) |
|  |  |  | *Hibiscus* | *Hibiscus sp.* | 3% (1/36) | 0% (0/1) | 0% (0/1) |
|  |  | *Solanaceae* | *Capsicum* | *Capsicum annuum* | 50% (4/8) | 50% (2/4) | 0% (0/4) |
|  |  |  |  | *Capsicum frutescens* | 92% (22/24) | 64% (14/22) | 0% (0/22) |
|  |  |  |  | **Subtotal 1** | **54% (50/92)** | **42% (21/50)** | **6% (3/50)** |
|  | Uncultivated | *Amaranthaceae* | *Amaranthus* | *Amaranthus spinosus* | 33% (4/12) | 75% (3/4) | 0% (0/4) |
|  |  |  |  | *Amaranthus viridis* | 8% (4/50) | 50% (2/4) | 0% (0/4) |
|  |  | *Asteraceae* | *Ageratum* | *Ageratum conyzoides* | 33% (3/9) | 33% (1/3) | 0% (0/3) |
|  |  | *Boraginaceae* | *Heliotropium* | *Heliotropium indicum* | 0% (0/7) | -% (-/0) | - |
|  |  | *Convolvulaceae* | *Ipomoea* | *Ipomoea alba* | 100% (8/8) | 75% (6/8) | 0% (0/8) |
|  |  | *Euphorbiaceae* | *Euphorbia* | *Euphorbia hirta* | 10% (4/41) | 0% (0/4) | 0% (0/4) |
|  |  | *Lamiaceae* | *Leucas* | *Leucas martinicens* | 30% (3/10) | 0% (0/3) | 0% (0/3) |
|  |  | *Malvaceae* | *Melochia* | *Melochia sp.* | 0% (0/3) | - | - |
|  |  |  | *Sida* | *Sida acuta* | 80% (20/25) | 35% (7/20) | 10% (2/20) |
|  |  |  |  | *Sida sp.* | 25% (3/12) | 67% (2/3) | 0% (0/3) |
|  |  | *Nyctaginaceae* | *Boerhavia* | *Boerhavia erecta* | 3% (2/59) | 0% (0/2) | 0% (0/2) |
|  |  | *Rubiaceae* | *Mitracarpus* | *Mitracarpus sp.* | 0% (0/9) | - | - |
|  |  |  |  | *Mitracarpus villosus* | 0% (0/35) | - | - |
|  |  | *Solanaceae* | *Physalis* | *Physalis ixocarpa* | 7% (1/15) | 0% (0/1) | 0% (0/1) |
|  |  | Undetermined | Undetermined | Undetermined | 0% (0/8) | - | - |
|  |  |  |  | **Subtotal 2** | **17% (52/303)** | **40% (21/52)** | **4% (2/52)** |
|  |  |  |  | **Subtotal 3** | **26% (102/395)** | **41% (42/102)** | **5% (5/102)** |
| Léguéma | Cultivated | *Malvaceae* | *Abelmoschus* | *Abelmoschus esculentus* | 7% (2/27) | 100% (2/2) | 0% (0/2) |
|  |  | *Solanaceae* | *Capsicum* | *Capsicum frutescens* | 0% (0/2) | - | - |
|  |  |  | *Solanum* | *Solanum lycopersicum* | 71% (30/42) | 70% (21/30) | 10% (3/30) |
|  |  |  |  | **Subtotal 1** | **45% (32/71)** | **72% (23/32)** | **9% (3/32)** |
|  | Uncultivated | *Amaranthaceae* | *Amaranthus* | *Amaranthus hybridus* | 0% (0/1) | - | - |
|  |  |  |  | *Amaranthus spinosus* | 5% (2/42) | 50% (1/2) | 0% (0/2) |
| Léguéma | Uncultivated | *Amaranthaceae* | *Amaranthus* | *Amaranthus viridis* | 4% (2/49) | 50% (1/2) | 0% (0/2) |
|  |  | *Asteraceae* | *Ageratum* | *Ageratum conyzoides* | 23% (13/56) | 85% (11/13) | 0% (0/13) |
|  |  | *Convolvulaceae* | *Ipomoea* | *Ipomoea alba* | 28% (7/25) | 29% (2/7) | 0% (0/7) |
|  |  | *Euphorbiaceae* | *Euphorbia* | *Euphorbia heterophylla* | 5% (2/38) | 50% (1/2) | 0% (0/2) |
|  |  |  |  | *Euphorbia hirta* | 2% (1/45) | 100% (1/1) | 0% (0/1) |
|  |  | *Hydroleaceae* | *Hydrolea* | *Hydrolea sp.* | 0% (0/3) | - | - |
|  |  | *Lamiaceae* | *Leucas* | *Leucas martinicens* | 13% (2/16) | 50% (1/2) | 0% (0/2) |
|  |  | *Malvaceae* | *Melochia* | *Melochia sp.* | 0% (0/2) | - | - |
|  |  |  | *Sida* | *Sida acuta* | 42% (27/64) | 67% (18/27) | 11% (3/27) |
|  |  |  |  | *Sida rhombifolia* | 14% (1/7) | 100% (1/1) | 0% (0/1) |
|  |  | *Nyctaginaceae* | *Boerhavia* | *Boerhavia erecta* | 5% (2/41) | 100% (2/2) | 0% (0/2) |
|  |  | *Pedaliaceae* | *Sesamum* | *Sesamum sp.* | 0% (0/2) | - | - |
|  |  | *Rubiaceae* | *Mitracarpus* | *Mitracarpus villosus* | 0% (0/4) | - | - |
|  |  | *Solanaceae* | *Physalis* | *Physalis ixocarpa* | 0% (0/19) | - | - |
|  |  |  |  | **Subtotal 2** | **14% (59/414)** | **66% (39/59)** | **5% (3/59)** |
|  |  |  |  | **Subtotal 3** | **19% (91/485)** | **68% (62/91)** | **7% (6/91)** |
|  |  |  |  | **Total** | **22% (193/880)** | **54% (104/193)** | **6% (11/193)** |

^$^Proportion of infected plants with positive status base on illmina sequencing method

^£^Proportion of infected whith full genome sequences obtained using the RCA-MinION and RCA-RFLP methods

subtotal 1 indicates the summary of infection rates for cultivated plants;

subtotal 2 indicates the summary of infection rates for uncultivated plants;

subtotal 3 indicates the summary of infection rates per localities.

**Supplementary Table 2: Summary of the sequenced CRESS DNA virus species in this study using either RCA-MinION or RCA-RFLP procedures**

ALCuCMB: ageratum leaf curl Cameroon betasatellite, BeAB: boerhavia erecta associated betasatellite, BeAV: boerhavia erecta associated virus, CLCuBFB: cotton leaf curl Burkina Faso betasatellite, CLCuCMA: cotton leaf curl Cameroon alphasatellite, CLCuGeB: cotton leaf curl Gezira betasatellite, CLCuGeV: cotton leaf curl Gezira virus, CleLCrV: cleome leaf crumple virus, CoLCGA: cotton leaf curl Gezira alphasatellite, CpCDV: chickpea chlorotic dwarf virus, IMLCV: ipomoea mild leaf curl virus, IYCrV: ipomoea yellow crinkle virus, OVTA: okra vein thickening alphasatellite, OVTV: okra vein thickening virus, OYCrCMA: okra yellow crinkle Cameroon alphasatellite, PepYVMLV:pepper yellow vein Mali virus, SaAB: sida acuta associated betasatellite, SaYLCA: sida acuta yellow leaf curl alphasatellite, SmaA: sorghum mastrevirus associated alphasatellite, SPLCV: sweet potato leaf curl virus, SYMBFV: sida yellow mosaic Burkina Faso virus, ToCSV: tomato curly stunt virus, ToLCBFV: tomato leaf curl Burkina Faso virus, ToLCCMV: tomato leaf curl Cameroon virus, ToLCGHV: tomato leaf curl Ghana virus, ToLCMLV: tomato leaf curl Mali virus, ToLCNaV: tomato leaf curl Namakely virus, ToLCNGV: tomato leaf curl Nigeria virus, ToLCuTGB: tomato leaf curl Togo betasatellite, ToLCuTGB: tomato leaf curl Togo betasatellite, ToVTV: tomato vein thickening virus,

| **Accession** | **Sequence ID** | **Isolation locality** | **Virus species** | **Virus common name** | **Family** | **Closest virus** | **Disimilarity rate** | **Virus Cluster** | **Sequence length** | **Host** |
| --- | --- | --- | --- | --- | --- | --- | --- | --- | --- | --- |
| **RCA-MinION** | |  |  |  |  |  |  |  |  |  |
| PX352841 | BF09102020_1_B_1 | Leguema | *Begomovirus capsicummaliense* | PepYVMLV | Geminiviridae | MH778651 PepYVMLV | 0.008 | A1 | 2790 | *Solanum lycopersicum* |
| PX352843 | BF09102020_8_D_2 | Leguema | *Begomovirus capsicummaliense* | PepYVMLV | Geminiviridae | FM876850 PepYVMLV | 0.011 | A1 | 2777 | *Solanum lycopersicum* |
| PX352854 | BF114_5_B_1 | Goue | *Begomovirus capsicummaliense* | PepYVMLV | Geminiviridae | FM876850 PepYVMLV | 0.038 | A1 | 2802 | *Capsicum annuum* |
| PX352851 | BF114_6_B_1 | Goue | *Begomovirus capsicummaliense* | PepYVMLV | Geminiviridae | FM876850 PepYVMLV | 0.01 | A1 | 2790 | *Capsicum annuum* |
| PX352857 | BF12_1_B_3 | Leguema | *Begomovirus capsicummaliense* | PepYVMLV | Geminiviridae | MH778686 PepYVMLV | 0.016 | A1 | 2788 | *Solanum lycopersicum* |
| PX352859 | BF12_1_B_5 | Leguema | *Begomovirus capsicummaliense* | PepYVMLV | Geminiviridae | MH778669 PepYVMLV | 0.06 | A1 | 2776 | *Solanum lycopersicum* |
| PX352845 | BF12_8_B_1 | Leguema | *Begomovirus capsicummaliense* | PepYVMLV | Geminiviridae | FM876850 PepYVMLV | 0.01 | A1 | 2789 | *Solanum lycopersicum* |
| PX352846 | BF126_1_B_2 | Goue | *Begomovirus capsicummaliense* | PepYVMLV | Geminiviridae | FM876850 PepYVMLV | 0.013 | A1 | 2787 | *Capsicum frutescens* |
| PX352847 | BF126_2_B_3 | Goue | *Begomovirus capsicummaliense* | PepYVMLV | Geminiviridae | FM876850 PepYVMLV | 0.012 | A1 | 2792 | *Capsicum frutescens* |
| PX352849 | BF126_7_B_2 | Goue | *Begomovirus capsicummaliense* | PepYVMLV | Geminiviridae | FM876850 PepYVMLV | 0.011 | A1 | 2781 | *Capsicum frutescens* |
| PX352856 | BF13102020_9_B_3 | Goue | *Begomovirus capsicummaliense* | PepYVMLV | Geminiviridae | FM876850 PepYVMLV | 0.032 | A1 | 1206 | *Capsicum frutescens* |
| PX352848 | BF138_12_B_1 | Goue | *Begomovirus capsicummaliense* | PepYVMLV | Geminiviridae | FM876850 PepYVMLV | 0.01 | A1 | 2792 | *Capsicum frutescens* |
| PX352842 | BF138_3_B_2 | Goue | *Begomovirus capsicummaliense* | PepYVMLV | Geminiviridae | MH778651 PepYVMLV | 0.009 | A1 | 2787 | *Amaranthus spinosus* |
| PX352855 | BF138_8_B_2 | Goue | *Begomovirus capsicummaliense* | PepYVMLV | Geminiviridae | FM876850 PepYVMLV | 0.035 | A1 | 2797 | *Capsicum annuum* |
| PX352852 | BF138_9_B_1 | Goue | *Begomovirus capsicummaliense* | PepYVMLV | Geminiviridae | FM876850 PepYVMLV | 0.012 | A1 | 2784 | *Capsicum annuum* |
| PX352858 | BF24_2_D_4 | Leguema | *Begomovirus capsicummaliense* | PepYVMLV | Geminiviridae | MH778686 PepYVMLV | 0.015 | A1 | 2791 | *Solanum lycopersicum* |
| PX352844 | BF24_3_D_3 | Leguema | *Begomovirus capsicummaliense* | PepYVMLV | Geminiviridae | FM876850 PepYVMLV | 0.01 | A1 | 2780 | *Solanum lycopersicum* |
| PX352850 | BF24_4_B_3 | Leguema | *Begomovirus capsicummaliense* | PepYVMLV | Geminiviridae | MH778651 PepYVMLV | 0.011 | A1 | 2796 | *Amaranthus viridis* |
| PX352853 | BF72_4_D_2 | Goue | *Begomovirus capsicummaliense* | PepYVMLV | Geminiviridae | FM876850 PepYVMLV | 0.01 | A1 | 2779 | *Capsicum frutescens* |
| PX352860 | BF09102020_10_B_1 | Leguema | *Begomovirus solanummaliense* | ToLCMLV | Geminiviridae | MH794643 ToLCMLV | 0.014 | A2 | 2814 | *Solanum lycopersicum* |
| PX352861 | BF09102020_8_B_2 | Leguema | *Begomovirus solanummaliense* | ToLCMLV | Geminiviridae | MH794643 ToLCMLV | 0.017 | A2 | 2773 | *Solanum lycopersicum* |
| PX352862 | BF09102020_9_B_1 | Leguema | *Begomovirus solanummaliense* | ToLCMLV | Geminiviridae | MH794643 ToLCMLV | 0.015 | A2 | 2780 | *Solanum lycopersicum* |
| PX352872 | BF12_1_B_6 | Leguema | *Begomovirus solanummaliense* | ToLCMLV | Geminiviridae | MH794643 ToLCMLV | 0.017 | A2 | 2771 | *Solanum lycopersicum* |
| PX352877 | BF12_1_B_7 | Leguema | *Begomovirus solanummaliense* | ToLCMLV | Geminiviridae | MH794643 ToLCMLV | 0.065 | A2 | 2785 | *Solanum lycopersicum* |
| PX352875 | BF12_3_B_1 | Leguema | *Begomovirus solanummaliense* | ToLCMLV | Geminiviridae | MH794643 ToLCMLV | 0.02 | A2 | 2855 | *Solanum lycopersicum* |
| PX352864 | BF12_5_B_2 | Leguema | *Begomovirus solanummaliense* | ToLCMLV | Geminiviridae | MH794643 ToLCMLV | 0.017 | A2 | 2779 | *Solanum lycopersicum* |
| PX352863 | BF12_6_B_6 | Leguema | *Begomovirus solanummaliense* | ToLCMLV | Geminiviridae | MH794643 ToLCMLV | 0.017 | A2 | 2777 | *Solanum lycopersicum* |
| PX352874 | BF24_1_B_1 | Leguema | *Begomovirus solanummaliense* | ToLCMLV | Geminiviridae | MH794643 ToLCMLV | 0.016 | A2 | 2805 | *Solanum lycopersicum* |
| PX352876 | BF24_1_B_3 | Leguema | *Begomovirus solanummaliense* | ToLCMLV | Geminiviridae | MH794643 ToLCMLV | 0.049 | A2 | 1045 | *Solanum lycopersicum* |
| PX352867 | BF24_12_B_2 | Leguema | *Begomovirus solanummaliense* | ToLCMLV | Geminiviridae | MH794643 ToLCMLV | 0.016 | A2 | 2778 | *Solanum lycopersicum* |
| PX352868 | BF24_2_B_1 | Leguema | *Begomovirus solanummaliense* | ToLCMLV | Geminiviridae | MH794643 ToLCMLV | 0.017 | A2 | 2770 | *Solanum lycopersicum* |
| PX352871 | BF24_3_B_1 | Leguema | *Begomovirus solanummaliense* | ToLCMLV | Geminiviridae | MH794643 ToLCMLV | 0.016 | A2 | 2805 | *Solanum lycopersicum* |
| PX352873 | BF24_4_B_7 | Leguema | *Begomovirus solanummaliense* | ToLCMLV | Geminiviridae | MH794643 ToLCMLV | 0.017 | A2 | 2784 | *Amaranthus viridis* |
| PX352865 | BF36_1_B_2 | Leguema | *Begomovirus solanummaliense* | ToLCMLV | Geminiviridae | MH794643 ToLCMLV | 0.017 | A2 | 2776 | *Solanum lycopersicum* |
| PX352866 | BF36_2_B_4 | Leguema | *Begomovirus solanummaliense* | ToLCMLV | Geminiviridae | MH794643 ToLCMLV | 0.018 | A2 | 2778 | *Solanum lycopersicum* |
| PX352870 | BF48_10_B_2 | Leguema | *Begomovirus solanummaliense* | ToLCMLV | Geminiviridae | MH794643 ToLCMLV | 0.017 | A2 | 2785 | *Solanum lycopersicum* |
| PX352869 | BF48_11_B_1 | Leguema | *Begomovirus solanummaliense* | ToLCMLV | Geminiviridae | MH794643 ToLCMLV | 0.017 | A2 | 2768 | *Solanum lycopersicum* |
| PX352879 | BF09102020_2_B_2 | Leguema | *Begomovirus solanumghanaense* | ToLCGHV | Geminiviridae | EU847739 ToLCGHV | 0.029 | A3 | 2800 | *Ageratum conyzoides* |
| PX352888 | BF09102020_7_B_3 | Leguema | *Begomovirus solanumghanaense* | ToLCGHV | Geminiviridae | EU847739 ToLCGHV | 0.044 | A3 | 1338 | *Boerhavia erecta* |
| PX352885 | BF12_11_B_2 | Leguema | *Begomovirus solanumghanaense* | ToLCGHV | Geminiviridae | HE659517 ToLCGHV | 0.073 | A3 | 2801 | *Ageratum conyzoides* |
| PX352886 | BF12_4_B_2 | Leguema | *Begomovirus solanumghanaense* | ToLCGHV | Geminiviridae | HE659517 ToLCGHV | 0.073 | A3 | 2800 | *Ageratum conyzoides* |
| PX352882 | BF12_6_B_2 | Leguema | *Begomovirus solanumghanaense* | ToLCGHV | Geminiviridae | EU847739 ToLCGHV | 0.027 | A3 | 2789 | *Solanum lycopersicum* |
| PX352884 | BF12_7_B_1 | Leguema | *Begomovirus solanumghanaense* | ToLCGHV | Geminiviridae | EU847739 ToLCGHV | 0.032 | A3 | 2753 | *Ageratum conyzoides* |
| PX352883 | BF24_8_B_1 | Leguema | *Begomovirus solanumghanaense* | ToLCGHV | Geminiviridae | EU847739 ToLCGHV | 0.044 | A3 | 2810 | *Euphorbia heterophylla* |
| PX352887 | BF36_7_B_2 | Leguema | *Begomovirus solanumghanaense* | ToLCGHV | Geminiviridae | HE659517 ToLCGHV | 0.073 | A3 | 2800 | *Ageratum conyzoides* |
| PX352889 | BF48_5_B_3 | Leguema | *Begomovirus solanumghanaense* | ToLCGHV | Geminiviridae | EU847739 ToLCGHV | 0.044 | A3 | 1342 | *Boerhavia erecta* |
| PX352881 | BF48_6_B_1 | Leguema | *Begomovirus solanumghanaense* | ToLCGHV | Geminiviridae | EU847739 ToLCGHV | 0.029 | A3 | 2804 | *Ageratum conyzoides* |
| PX352880 | BF48_9_B_2 | Leguema | *Begomovirus solanumghanaense* | ToLCGHV | Geminiviridae | EU847739 ToLCGHV | 0.03 | A3 | 2803 | *Ageratum conyzoides* |
| PX352913 | BF625-714_3_B_1 | Leguema | *Begomovirus solanumghanaense* | ToLCGHV | Geminiviridae | EU847739 ToLCGHV | 0.073 | A3 | 1004 | *Solanum lycopersicum* |
| PX352894 | BF09102020_4_B_1 | Leguema | *Begomovirus sidacutae* | SaYLCV | Geminiviridae | MH794661 SaYLCV | 053 | A4 | 2763 | *Sida acuta* |
| PX352899 | BF102_NOTHAC_1_B_1 | Leguema | *Begomovirus sidacutae* | SaYLCV | Geminiviridae | MH794661 SaYLCV | 06 | A4 | 2764 | *Sida acuta* |
| PX352897 | BF24_7_B_4 | Leguema | *Begomovirus sidacutae* | SaYLCV | Geminiviridae | MH794661 SaYLCV | 05 | A4 | 2771 | *Sida acuta* |
| PX352896 | BF24_9_B_1 | Leguema | *Begomovirus sidacutae* | SaYLCV | Geminiviridae | MH794661 SaYLCV | 054 | A4 | 2765 | *Sida acuta* |
| PX352895 | BF36_8_B_3 | Leguema | *Begomovirus sidacutae* | SaYLCV | Geminiviridae | MH794661 SaYLCV | 054 | A4 | 2773 | *Sida acuta* |
| PX352898 | BF72_12_B_3 | Goue | *Begomovirus sidacutae* | SaYLCV | Geminiviridae | MH794661 SaYLCV | 048 | A4 | 2770 | *Sida acuta* |
| PX352916 | BF09102020_5_B_1 | Leguema | *Begomovirus ipomoeae* | SPLCV | Geminiviridae | KY270781 SPLCV | 0.078 | A5 | 2857 | *Ipomea alba* |
| PX352918 | BF102_NOTHAC_4_B_1 | Leguema | *Begomovirus ipomoeae* | SPLCV | Geminiviridae | FR751068 SPLCV | 021 | A5 | 1294 | *Ipomea alba* |
| PX352917 | BF36_6_B_2 | Leguema | *Begomovirus ipomoeae* | SPLCV | Geminiviridae | KY270781 SPLCV | 0.082 | A5 | 2852 | *Ipomea alba* |
| PX352914 | BF09102020_7_B_2 | Leguema | *Begomovirus boerhaviae* | BeAV 1 | Geminiviridae | KT382327 ToLCCMV | 048 | A6 | 1239 | *Boerhavia erecta* |
| PX352915 | BF48_5_B_4 | Leguema | *Begomovirus boerhaviae* | BeAV 1 | Geminiviridae | KT382327 ToLCCMV | 035 | A6 | 1236 | *Boerhavia erecta* |
| PX352900 | BF114_10_B_2 | Leguema | *Begomovirus gossypigeziraense* | CLCuGeV | Geminiviridae | MH794664 CLCuGeV | 0.026 | A7 | 2748 | *Sida acuta* |
| PX352906 | BF24_10_B_2 | Leguema | *Begomovirus gossypigeziraense* | CLCuGeV | Geminiviridae | MH794662 CLCuGeV | 0.016 | A7 | 1369 | *Sida acuta* |
| PX352901 | BF24_10_B_5 | Leguema | *Begomovirus gossypigeziraense* | CLCuGeV | Geminiviridae | MH794661 CLCuGeV | 0.038 | A7 | 2771 | *Sida acuta* |
| PX352904 | BF36_3_B_2 | Leguema | *Begomovirus gossypigeziraense* | CLCuGeV | Geminiviridae | MN372225 CLCuGeV | 0.098 | A7 | 564 | *Sida rhombifolia* |
| PX352902 | BF36_4_B_3 | Leguema | *Begomovirus gossypigeziraense* | CLCuGeV | Geminiviridae | MH794654 CLCuGeV | 0.017 | A7 | 2769 | *Sida acuta* |
| PX352912 | BF72_12_B_4 | Goue | *Begomovirus gossypigeziraense* | CLCuGeV | Geminiviridae | MH794666 CLCuGeV | 0.079 | A7 | 1707 | *Sida acuta* |
| PX352905 | BF780-888_5_B_2 | Goue | *Begomovirus gossypigeziraense* | CLCuGeV | Geminiviridae | MN372225 CLCuGeV | 001 | A7 | 708 | *Sida acuta* |
| PX352903 | BF90_NOTHAC_3_B_3 | Goue | *Begomovirus gossypigeziraense* | CLCuGeV | Geminiviridae | MN372225 CLCuGeV | 0.013 | A7 | 1269 | *Sida acuta* |
| PX352891 | BF12_12_B_1 | Leguema | *Begomovirus boerectae* | BeAV 2 | Geminiviridae | FJ685621 ToLCNGV | 038 | A8 | 2787 | *Boerhavia erecta* |
| PX352892 | BF48_12_B_1 | Leguema | *Begomovirus boerectae* | BeAV 2 | Geminiviridae | FJ685621 ToLCNGV | 037 | A8 | 2779 | *Solanum lycopersicum* |
| PX352919 | BF126_10_B_1 | Goue | *Begomovirus ipomogouense* | IYCrV | Geminiviridae | MH778695 PepYVMLV | 0.368 | A9 | 2800 | *Ipomea alba* |
| PX352923 | BF126_8_B_1 | Goue | *Begomovirus ipomogouense* | IYCrV | Geminiviridae | FM876850 PepYVMLV | 0.356 | A9 | 2821 | *Ipomea alba* |
| PX352924 | BF126_9_B_3 | Goue | *Begomovirus ipomogouense* | IYCrV | Geminiviridae | KX853168 ToLCBFV | 0.361 | A9 | 2823 | *Ipomea alba* |
| PX352920 | BF138_1_B_1 | Goue | *Begomovirus ipomogouense* | IYCrV | Geminiviridae | MH778669 PepYVMLV | 0.364 | A9 | 2814 | *Ipomea alba* |
| PX352921 | BF138_2_B_2 | Goue | *Begomovirus ipomogouense* | IYCrV | Geminiviridae | MH778669 PepYVMLV | 0.364 | A9 | 2815 | *Ipomea alba* |
| PX352922 | BF90_NOTHAC_10_B_1 | Goue | *Begomovirus ipomogouense* | IYCrV | Geminiviridae | MH778669 PepYVMLV | 0.367 | A9 | 2818 | *Ipomea alba* |
| PX352907 | BF126_10_B_2 | Goue | *Begomovirus ipomoalbae* | IMLCV | Geminiviridae | FN600540 ToLCNaV | 0.314 | A10 | 2825 | *Ipomea alba* |
| PX352908 | BF126_11_B_1 | Goue | *Begomovirus ipomoalbae* | IMLCV | Geminiviridae | FN600540 ToLCNaV | 0.313 | A10 | 2820 | *Ipomea alba* |
| PX352910 | BF126_9_B_1 | Goue | *Begomovirus ipomoalbae* | IMLCV | Geminiviridae | FN600540 ToLCNaV | 0.311 | A10 | 2817 | *Ipomea alba* |
| PX352911 | BF138_1_B_3 | Goue | *Begomovirus ipomoalbae* | IMLCV | Geminiviridae | FN600540 ToLCNaV | 0.311 | A10 | 2819 | *Ipomea alba* |
| PX352909 | BF138_2_B_1 | Goue | *Begomovirus ipomoalbae* | IMLCV | Geminiviridae | FN600540 ToLCNaV | 0.311 | A10 | 2822 | *Ipomea alba* |
| PX352878 | BF48_3_B_1 | Leguema | *Begomovirus solanumghanaense* | ToLCCMV | Geminiviridae | KT382327 ToLCCMV | 0.085 | A11 | 2801 | *Ageratum conyzoides* |
| PX352925 | BF625-714_11_B_4 | Goue | *Begomovirus sidafasoense* | SYMBFV | Geminiviridae | OK813889 ToCSV | 0.332 | A12 | 2734 | *Sida sp* |
| PX352926 | BF625-714_11_B_5 | Goue | *Begomovirus sidafasoense* | SYMBFV | Geminiviridae | OK813889 ToCSV | 0.313 | A12 | 2776 | *Sida sp* |
| PX352893 | BF985-1126_10_B_2 | Goue | *Begomovirus abelmosentus* | OVTV | Geminiviridae | FJ469626 CLCuGeV | 017 | A13 | 681 | *Abelmoschus esculentus* |
| PX352890 | BF12_6_B_7 | Leguema | *Begomovirus tomatosicum* | ToVTV | Geminiviridae | EU847739 ToLCGHV | 022 | A14 | 2820 | *Solanum lycopersicum* |
| PX352833 | BF114_3_B_1 | Goue | *Mastrevirus cicerparvi* | CpCDV | Geminiviridae | KY817246 CpCDV | 0.014 | M1 | 2575 | *Amaranthus spinosus* |
| PX352834 | BF13102020_3_B_2 | Leguema | *Mastrevirus cicerparvi* | CpCDV | Geminiviridae | KY817246 CpCDV | 0.015 | M1 | 1372 | *Abelmoschus esculentus* |
| PX352840 | BF138_3_B_1 | Goue | *Mastrevirus cicerparvi* | CpCDV | Geminiviridae | AM933135 CpCDV | 0.26 | M1 | 1395 | *Amaranthus spinosus* |
| PX352837 | BF138_4_B_1 | Goue | *Mastrevirus cicerparvi* | CpCDV | Geminiviridae | KC172676 CpCDV | 0.21 | M1 | 2598 | *Amaranthus viridis* |
| PX352839 | BF138_5_B_1 | Goue | *Mastrevirus cicerparvi* | CpCDV | Geminiviridae | KC172676 CpCDV | 0.21 | M1 | 2599 | *Amaranthus viridis* |
| PX352835 | BF138_6_B_1 | Goue | *Mastrevirus cicerparvi* | CpCDV | Geminiviridae | KY817246 CpCDV | 0.022 | M1 | 1427 | *Amaranthus spinosus* |
| PX352838 | BF138_7_B_1 | Goue | *Mastrevirus cicerparvi* | CpCDV | Geminiviridae | KC172676 CpCDV | 0.211 | M1 | 2599 | *Amaranthus spinosus* |
| PX352836 | BF625-714_7_B_1 | Leguema | *Mastrevirus cicerparvi* | CpCDV | Geminiviridae | KY047532 CpCDV | 0.081 | M1 | 123 | *Leucas martinicens* |
| PX316524 | BF09102020_1_B_2 | Leguema | *Begomovirus capsicummaliense* | PepYVMLV | Geminiviridae | MK092765 PepYVMLV | 0.009 | B1 | 2668 | *Solanum lycopersicum* |
| PX316527 | BF114_5_B_2 | Goue | *Begomovirus capsicummaliense* | PepYVMLV | Geminiviridae | MK092756 PepYVMLV | 0.024 | B1 | 2674 | *Capsicum annuum* |
| PX316531 | BF114_6_B_3 | Goue | *Begomovirus capsicummaliense* | PepYVMLV | Geminiviridae | MK092752 PepYVMLV | 0.012 | B1 | 2667 | *Capsicum annuum* |
| PX316544 | BF114_9_B_1 | Goue | *Begomovirus capsicummaliense* | PepYVMLV | Geminiviridae | MK092748 PepYVMLV | 0.044 | B1 | 2672 | *Capsicum frutescens* |
| PX316526 | BF12_8_B_2 | Leguema | *Begomovirus capsicummaliense* | PepYVMLV | Geminiviridae | MK092765 PepYVMLV | 0.014 | B1 | 2673 | *Solanum lycopersicum* |
| PX316532 | BF126_1_B_1 | Goue | *Begomovirus capsicummaliense* | PepYVMLV | Geminiviridae | MK092752 PepYVMLV | 0.006 | B1 | 2666 | *Capsicum frutescens* |
| PX316535 | BF126_2_B_2 | Goue | *Begomovirus capsicummaliense* | PepYVMLV | Geminiviridae | MK092752 PepYVMLV | 0.022 | B1 | 2673 | *Capsicum frutescens* |
| PX316534 | BF126_3_B_1 | Goue | *Begomovirus capsicummaliense* | PepYVMLV | Geminiviridae | MK092752 PepYVMLV | 0.019 | B1 | 2671 | *Capsicum frutescens* |
| PX316533 | BF126_4_B_1 | Goue | *Begomovirus capsicummaliense* | PepYVMLV | Geminiviridae | MK092752 PepYVMLV | 0.006 | B1 | 2670 | *Capsicum frutescens* |
| PX316543 | BF126_5_B_1 | Goue | *Begomovirus capsicummaliense* | PepYVMLV | Geminiviridae | MK092752 PepYVMLV | 0.051 | B1 | 2678 | *Capsicum frutescens* |
| PX316537 | BF126_7_B_1 | Goue | *Begomovirus capsicummaliense* | PepYVMLV | Geminiviridae | MK092752 PepYVMLV | 0.018 | B1 | 2673 | *Capsicum frutescens* |
| PX316541 | BF13102020_11_B_1 | Goue | *Begomovirus capsicummaliense* | PepYVMLV | Geminiviridae | MH460533 PepYVMLV | 0.035 | B1 | 2704 | *Capsicum frutescens* |
| PX316542 | BF13102020_5_B_1 | Goue | *Begomovirus capsicummaliense* | PepYVMLV | Geminiviridae | MH460533 PepYVMLV | 0.026 | B1 | 2674 | *Capsicum frutescens* |
| PX316529 | BF13102020_7_B_1 | Goue | *Begomovirus capsicummaliense* | PepYVMLV | Geminiviridae | MK092765 PepYVMLV | 0.017 | B1 | 2676 | *Capsicum frutescens* |
| PX316540 | BF13102020_9_B_2 | Goue | *Begomovirus capsicummaliense* | PepYVMLV | Geminiviridae | MK092752 PepYVMLV | 0.029 | B1 | 2679 | *Capsicum frutescens* |
| PX316545 | BF138_12_B_2 | Goue | *Begomovirus capsicummaliense* | PepYVMLV | Geminiviridae | MK092748 PepYVMLV | 0.043 | B1 | 2674 | *Capsicum frutescens* |
| PX316546 | BF138_3_B_3 | Goue | *Begomovirus capsicummaliense* | PepYVMLV | Geminiviridae | MK092748 PepYVMLV | 0.046 | B1 | 2672 | *Amaranthus spinosus* |
| PX316528 | BF138_8_B_1 | Goue | *Begomovirus capsicummaliense* | PepYVMLV | Geminiviridae | MK092756 PepYVMLV | 0.023 | B1 | 2678 | *Capsicum annuum* |
| PX316536 | BF138_9_B_2 | Goue | *Begomovirus capsicummaliense* | PepYVMLV | Geminiviridae | MK092752 PepYVMLV | 0.008 | B1 | 2684 | *Capsicum annuum* |
| PX316525 | BF24_4_B_2 | Leguema | *Begomovirus capsicummaliense* | PepYVMLV | Geminiviridae | MK092765 PepYVMLV | 0.01 | B1 | 2669 | *Amaranthus viridis* |
| PX316530 | BF72_4_B_1 | Goue | *Begomovirus capsicummaliense* | PepYVMLV | Geminiviridae | MK092765 PepYVMLV | 0.02 | B1 | 2598 | *Capsicum frutescens* |
| PX316547 | BF72_5_B_1 | Goue | *Begomovirus capsicummaliense* | PepYVMLV | Geminiviridae | MK092748 PepYVMLV | 0.043 | B1 | 2668 | *Capsicum frutescens* |
| PX316548 | BF72_6_B_1 | Goue | *Begomovirus capsicummaliense* | PepYVMLV | Geminiviridae | MK092748 PepYVMLV | 0.043 | B1 | 2670 | *Ageratum conyzoides* |
| PX316538 | BF90_NOTHAC_1_B_1 | Goue | *Begomovirus capsicummaliense* | PepYVMLV | Geminiviridae | MK092752 PepYVMLV | 0.006 | B1 | 2672 | *Capsicum frutescens* |
| PX316539 | BF90_NOTHAC_4_B_1 | Goue | *Begomovirus capsicummaliense* | PepYVMLV | Geminiviridae | MK092752 PepYVMLV | 0.016 | B1 | 2681 | *Capsicum frutescens* |
| PX352938 | BF13102020_1_B_4 | Leguema | *Colecusatellite gossypigeziraense* | CoLCGA | Alphasatellitidae | MK032300 CoLCGA | 0.015 | a1 | 1391 | *Abelmoschus esculentus* |
| PX352939 | BF625-714_6_B_1 | Leguema | *Colecusatellite gossypigeziraense* | CoLCGA | Alphasatellitidae | MK032300 CoLCGA | 0.014 | a1 | 1394 | *Solanum lycopersicum* |
| PX352945 | BF780-888_5_B_3 | Goue | *Colecusatellite gossypigeziraense* | CoLCGA | Alphasatellitidae | EU589450 CoLCGA | 001 | a1 | 595 | *Sida acuta* |
| PX352940 | BF985-1126_6_B_4 | Goue | *Colecusatellite gossypigeziraense* | CoLCGA | Alphasatellitidae | FN554580 CoLCGA | 0.026 | a1 | 1393 | *Abelmoschus esculentus* |
| PX352941 | BF36_9_B_1 | Leguema | *Colecusatellite gossypigeziraensecundi* | CoLCGA 2 | Alphasatellitidae | KY652676 CoLCGA | 0.235 | a2 | 1383 | *Sida acuta* |
| PX352927 | BF102_NOTHAC_3_B_1 | Leguema | *Colecusatellite sidacutae* | SaYLCA | Alphasatellitidae | HM446369 CoLCGA | 066 | a3 | 1401 | *Sida acuta* |
| PX352928 | BF114_11_B_4 | Leguema | *Colecusatellite sidacutae* | SaYLCA | Alphasatellitidae | HM446369 CoLCGA | 069 | a3 | 1391 | *Sida acuta* |
| PX352935 | BF114_12_B_2 | Leguema | *Colecusatellite sidacutae* | SaYLCA | Alphasatellitidae | MK032301 CoLCGA | 07 | a3 | 1390 | *Sida acuta* |
| PX352934 | BF13102020_4_B_1 | Leguema | *Colecusatellite sidacutae* | SaYLCA | Alphasatellitidae | HM446369 CoLCGA | 076 | a3 | 1406 | *Sida acuta* |
| PX352942 | BF210122-946-1149_2_B_2 | Leguema | *Colecusatellite sidacutae* | SaYLCA | Alphasatellitidae | KY652676 CoLCGA | 0.28 | a3 | 697 | *Sida acuta* |
| PX352936 | BF24_10_B_4 | Leguema | *Colecusatellite sidacutae* | SaYLCA | Alphasatellitidae | MK032300 CoLCGA | 069 | a3 | 1392 | *Sida acuta* |
| PX352937 | BF24_9_B_2 | Leguema | *Colecusatellite sidacutae* | SaYLCA | Alphasatellitidae | MK032301 CoLCGA | 028 | a3 | 1403 | *Sida acuta* |
| PX352944 | BF24_9_B_3 | Leguema | *Colecusatellite sidacutae* | SaYLCA | Alphasatellitidae | KY652676 CoLCGA | 0.285 | a3 | 708 | *Sida acuta* |
| PX352929 | BF36_10_B_4 | Leguema | *Colecusatellite sidacutae* | SaYLCA | Alphasatellitidae | HM446369 CoLCGA | 057 | a3 | 1388 | *Sida acuta* |
| PX352932 | BF36_2_B_3 | Leguema | *Colecusatellite sidacutae* | SaYLCA | Alphasatellitidae | HM446369 CoLCGA | 066 | a3 | 1397 | *Solanum lycopersicum* |
| PX352933 | BF36_8_B_1 | Leguema | *Colecusatellite sidacutae* | SaYLCA | Alphasatellitidae | HM446369 CoLCGA | 053 | a3 | 1394 | *Sida acuta* |
| PX352930 | BF36_9_B_4 | Leguema | *Colecusatellite sidacutae* | SaYLCA | Alphasatellitidae | HM446369 CoLCGA | 063 | a3 | 1367 | *Sida acuta* |
| PX352931 | BF72_12_B_1 | Goue | *Colecusatellite sidacutae* | SaYLCA | Alphasatellitidae | HE858192 CoLCGA | 039 | a3 | 1394 | *Sida acuta* |
| PX352943 | BF72_9_B_1 | Goue | *Colecusatellite sidacutae* | SaYLCA | Alphasatellitidae | MK032301 CoLCGA | 099 | a3 | 701 | *Sida acuta* |
| PX352955 | BF36_10_B_1 | Leguema | *Gosmusatellite gossypicameroonense* | CLCuCMA | Alphasatellitidae | FM164739 CLCuCMA | 006 | a4 | 1374 | *Sida acuta* |
| PX352956 | BF36_8_B_4 | Leguema | *Gosmusatellite gossypicameroonense* | CLCuCMA | Alphasatellitidae | FM164739 CLCuCMA | 002 | a4 | 1373 | *Sida acuta* |
| PX352954 | BF985-1126_10_B_3 | Goue | *Gosmusatellite abelmosentus* | OVTA | Alphasatellitidae | FN675286 OYCrCMA | 0.201 | a5 | 655 | *Abelmoschus esculentus* |
| PX352946 | BF114_11_B_2 | Leguema | *Gosmusatellite abelmoschuscameroonense* | OYCrCMA | Alphasatellitidae | FN675287 OYCrCMA | 0.026 | a6 | 1334 | *Sida acuta* |
| PX352952 | BF36_3_B_3 | Leguema | *Gosmusatellite abelmoschuscameroonense* | OYCrCMA | Alphasatellitidae | MK032295 CoLCGA | 0.033 | a6 | 1363 | *Sida rhombifolia* |
| PX352953 | BF36_4_B_2 | Leguema | *Gosmusatellite abelmoschuscameroonense* | OYCrCMA | Alphasatellitidae | MK032295 CoLCGA | 0.027 | a6 | 1360 | *Sida acuta* |
| PX352947 | BF72_10_B_1 | Goue | *Gosmusatellite abelmoschuscameroonense* | OYCrCMA | Alphasatellitidae | FN554581 CoLCGA | 0.025 | a6 | 1361 | *Sida acuta* |
| PX352951 | BF72_12_B_6 | Goue | *Gosmusatellite abelmoschuscameroonense* | OYCrCMA | Alphasatellitidae | MK032295 CoLCGA | 0.024 | a6 | 1365 | *Sida acuta* |
| PX352948 | BF72_9_B_2 | Goue | *Gosmusatellite abelmoschuscameroonense* | OYCrCMA | Alphasatellitidae | FN554581 CoLCGA | 0.024 | a6 | 1355 | *Sida acuta* |
| PX352949 | BF985-1126_5_B_5 | Goue | *Gosmusatellite abelmoschuscameroonense* | OYCrCMA | Alphasatellitidae | MK032295 CoLCGA | 0.001 | a6 | 1358 | *Abelmoschus esculentus* |
| PX352950 | BF985-1126_6_B_3 | Goue | *Gosmusatellite abelmoschuscameroonense* | OYCrCMA | Alphasatellitidae | MK032295 CoLCGA | 0 | a6 | 1358 | *Abelmoschus esculentus* |
| PX352957 | BF126_6_B_1 | Goue | *Unclassified* | CoLCGA 1 | Alphasatellitidae | MN901968 SmaA | 0.484 | a7 | 1370 | *Sida acuta* |
| PX352959 | BF72_12_B_2 | Goue | *Unclassified* | CoLCGA 1 | Alphasatellitidae | HE858192 CoLCGA | 0.484 | a7 | 1377 | *Sida acuta* |
| PX352958 | BF90_NOTHAC_3_B_1 | Goue | *Unclassified* | CoLCGA 1 | Alphasatellitidae | MN901968 SmaA | 0.482 | a7 | 1369 | *Sida acuta* |
| PX352782 | BF36_4_B_1 | Leguema | *Betasatellite gossypigeziraense* | CLCuGeB | Tolecusatellitidae | FN554574 CLCuGeB | 0.021 | b1 | 1354 | *Sida acuta* |
| PX352784 | BF90_NOTHAC_3_B_4 | Goue | *Betasatellite gossypigeziraense* | CLCuGeB | Tolecusatellitidae | MK032303 CLCuGeB | 0.069 | b1 | 1353 | *Sida acuta* |
| PX352783 | BF985-1126_6_B_1 | Goue | *Betasatellite gossypigeziraense* | CLCuGeB | Tolecusatellitidae | MK032303 CLCuGeB | 0.024 | b1 | 1355 | *Abelmoschus esculentus* |
| PX352812 | BF09102020_11_B_2 | Leguema | *Betasatellite boerhaviae* | BeAB | Tolecusatellitidae | FM164738 ALCuCMB | 093 | b2 | 1392 | *Ageratum conyzoides* |
| PX352813 | BF09102020_12_B_2 | Leguema | *Betasatellite boerhaviae* | BeAB | Tolecusatellitidae | FM164738 ALCuCMB | 093 | b2 | 1392 | *Ageratum conyzoides* |
| PX352814 | BF48_12_B_2 | Leguema | *Betasatellite boerhaviae* | BeAB | Tolecusatellitidae | FM164738 ALCuCMB | 095 | b2 | 1394 | *Solanum lycopersicum* |
| PX352816 | BF48_2_B_1 | Leguema | *Betasatellite boerhaviae* | BeAB | Tolecusatellitidae | FM164738 ALCuCMB | 094 | b2 | 1400 | *Sida acuta* |
| PX352815 | BF48_9_B_1 | Leguema | *Betasatellite boerhaviae* | BeAB | Tolecusatellitidae | FM164738 ALCuCMB | 095 | b2 | 1391 | *Ageratum conyzoides* |
| PX352817 | BF09102020_2_B_3 | Leguema | *Betasatellite solanighanaensetertii* | ToLCGHB 3 | Tolecusatellitidae | HQ586965 ToLCuTGB | 013 | b3 | 1400 | *Ageratum conyzoides* |
| PX352818 | BF09102020_7_B_1 | Leguema | *Betasatellite solanighanaensetertii* | ToLCGHB 3 | Tolecusatellitidae | HQ586965 ToLCuTGB | 015 | b3 | 1395 | *Boerhavia erecta* |
| PX352821 | BF12_7_B_2 | Leguema | *Betasatellite solanighanaensetertii* | ToLCGHB 3 | Tolecusatellitidae | HQ586965 ToLCuTGB | 013 | b3 | 1390 | *Ageratum conyzoides* |
| PX352822 | BF24_8_B_2 | Leguema | *Betasatellite solanighanaensetertii* | ToLCGHB 3 | Tolecusatellitidae | HQ586965 ToLCuTGB | 011 | b3 | 1396 | *Euphorbia heterophylla* |
| PX352823 | BF48_3_B_2 | Leguema | *Betasatellite solanighanaensetertii* | ToLCGHB 3 | Tolecusatellitidae | HQ586965 ToLCuTGB | 014 | b3 | 1395 | *Ageratum conyzoides* |
| PX352819 | BF48_5_B_1 | Leguema | *Betasatellite solanighanaensetertii* | ToLCGHB 3 | Tolecusatellitidae | HQ586965 ToLCuTGB | 015 | b3 | 1392 | *Boerhavia erecta* |
| PX352820 | BF48_6_B_2 | Leguema | *Betasatellite solanighanaensetertii* | ToLCGHB 3 | Tolecusatellitidae | HQ586965 ToLCuTGB | 014 | b3 | 1396 | *Ageratum conyzoides* |
| PX352825 | BF48_9_B_3 | Leguema | *Betasatellite solanighanaensetertii* | ToLCGHB 3 | Tolecusatellitidae | HQ586965 ToLCuTGB | 019 | b3 | 1363 | *Ageratum conyzoides* |
| PX352824 | BF625-714_3_B_2 | Leguema | *Betasatellite solanighanaensetertii* | ToLCGHB 3 | Tolecusatellitidae | HQ586965 ToLCuTGB | 014 | b3 | 1383 | *Solanum lycopersicum* |
| PX352785 | BF09102020_3_B_3 | Leguema | *Betasatellite gossypiburkinafasoense* | CLCuBFB | Tolecusatellitidae | MK032309 CLCuGeB | 0.045 | b4 | 1353 | *Amaranthus spinosus* |
| PX352786 | BF09102020_4_B_2 | Leguema | *Betasatellite gossypiburkinafasoense* | CLCuBFB | Tolecusatellitidae | MK032309 CLCuGeB | 0.046 | b4 | 1363 | *Sida acuta* |
| PX352788 | BF102_NOTHAC_1_B_2 | Leguema | *Betasatellite gossypiburkinafasoense* | CLCuBFB | Tolecusatellitidae | MK032309 CLCuGeB | 0.049 | b4 | 1360 | *Sida acuta* |
| PX352806 | BF102_NOTHAC_3_B_6 | Leguema | *Betasatellite gossypiburkinafasoense* | CLCuBFB | Tolecusatellitidae | MK032307 CLCuGeB | 0.065 | b4 | 1362 | *Sida acuta* |
| PX352791 | BF114_10_B_1 | Leguema | *Betasatellite gossypiburkinafasoense* | CLCuBFB | Tolecusatellitidae | MK032307 CLCuGeB | 0.043 | b4 | 1366 | *Sida acuta* |
| PX352799 | BF114_11_B_1 | Leguema | *Betasatellite gossypiburkinafasoense* | CLCuBFB | Tolecusatellitidae | MK032309 CLCuGeB | 0.045 | b4 | 1377 | *Sida acuta* |
| PX352801 | BF114_12_B_1 | Leguema | *Betasatellite gossypiburkinafasoense* | CLCuBFB | Tolecusatellitidae | MK032307 CLCuGeB | 0.052 | b4 | 1365 | *Sida acuta* |
| PX352798 | BF13102020_2_B_2 | Leguema | *Betasatellite gossypiburkinafasoense* | CLCuBFB | Tolecusatellitidae | MK032307 CLCuGeB | 0.046 | b4 | 1359 | *Sida acuta* |
| PX352789 | BF24_10_B_1 | Leguema | *Betasatellite gossypiburkinafasoense* | CLCuBFB | Tolecusatellitidae | MK032307 CLCuGeB | 0.041 | b4 | 1368 | *Sida acuta* |
| PX352805 | BF24_10_B_3 | Leguema | *Betasatellite gossypiburkinafasoense* | CLCuBFB | Tolecusatellitidae | MK032307 CLCuGeB | 0.073 | b4 | 1367 | *Sida acuta* |
| PX352796 | BF24_4_B_5 | Leguema | *Betasatellite gossypiburkinafasoense* | CLCuBFB | Tolecusatellitidae | MK032308 CLCuGeB | 0.059 | b4 | 1365 | *Amaranthus viridis* |
| PX352795 | BF24_6_B_2 | Leguema | *Betasatellite gossypiburkinafasoense* | CLCuBFB | Tolecusatellitidae | MK032307 CLCuGeB | 0.044 | b4 | 1361 | *Sida acuta* |
| PX352797 | BF24_7_B_2 | Leguema | *Betasatellite gossypiburkinafasoense* | CLCuBFB | Tolecusatellitidae | MK032308 CLCuGeB | 0.055 | b4 | 1364 | *Sida acuta* |
| PX352790 | BF24_9_B_5 | Leguema | *Betasatellite gossypiburkinafasoense* | CLCuBFB | Tolecusatellitidae | MK032307 CLCuGeB | 0.04 | b4 | 1365 | *Sida acuta* |
| PX352793 | BF36_10_B_7 | Leguema | *Betasatellite gossypiburkinafasoense* | CLCuBFB | Tolecusatellitidae | MK032307 CLCuGeB | 0.055 | b4 | 1366 | *Sida acuta* |
| PX352800 | BF36_11_D_2 | Leguema | *Betasatellite gossypiburkinafasoense* | CLCuBFB | Tolecusatellitidae | MK032307 CLCuGeB | 0.053 | b4 | 1355 | *Sida acuta* |
| PX352787 | BF36_8_B_2 | Leguema | *Betasatellite gossypiburkinafasoense* | CLCuBFB | Tolecusatellitidae | MK032309 CLCuGeB | 0.044 | b4 | 1358 | *Sida acuta* |
| PX352794 | BF625-714_6_B_2 | Leguema | *Betasatellite gossypiburkinafasoense* | CLCuBFB | Tolecusatellitidae | MK032307 CLCuGeB | 0.044 | b4 | 1367 | *Solanum lycopersicum* |
| PX352802 | BF72_10_B_2 | Goue | *Betasatellite gossypiburkinafasoense* | CLCuBFB | Tolecusatellitidae | MK032307 CLCuGeB | 0.043 | b4 | 1339 | *Sida acuta* |
| PX352792 | BF72_12_B_5 | Goue | *Betasatellite gossypiburkinafasoense* | CLCuBFB | Tolecusatellitidae | MK032307 CLCuGeB | 0.048 | b4 | 1367 | *Sida acuta* |
| PX352803 | BF72_8_B_1 | Goue | *Betasatellite gossypiburkinafasoense* | CLCuBFB | Tolecusatellitidae | MK032307 CLCuGeB | 0.047 | b4 | 1358 | *Sida acuta* |
| PX352804 | BF72_9_B_3 | Goue | *Betasatellite gossypiburkinafasoense* | CLCuBFB | Tolecusatellitidae | MK032307 CLCuGeB | 0.046 | b4 | 1356 | *Sida acuta* |
| PX352826 | BF12_11_B_1 | Leguema | *Betasatellite solanighanaensequarti* | ToLCGHB 4 | Tolecusatellitidae | FR717138 AgLCCMB | 031 | b5 | 1400 | *Ageratum conyzoides* |
| PX352830 | BF12_2_B_2 | Leguema | *Betasatellite solanighanaensequarti* | ToLCGHB 4 | Tolecusatellitidae | FR717138 ALCuCMB | 035 | b5 | 1381 | *Ageratum conyzoides* |
| PX352827 | BF12_4_B_1 | Leguema | *Betasatellite solanighanaensequarti* | ToLCGHB 4 | Tolecusatellitidae | FR717138 ALCuCMB | 03 | b5 | 1398 | *Ageratum conyzoides* |
| PX352829 | BF24_4_B_8 | Leguema | *Betasatellite solanighanaensequarti* | ToLCGHB 4 | Tolecusatellitidae | FR717138 ALCuCMB | 028 | b5 | 1390 | *Amaranthus viridis* |
| PX352831 | BF36_5_B_1 | Leguema | *Betasatellite solanighanaensequarti* | ToLCGHB 4 | Tolecusatellitidae | FR717138 ALCuCMB | 031 | b5 | 1392 | *Euphorbia hirta* |
| PX352828 | BF36_7_B_1 | Leguema | *Betasatellite solanighanaensequarti* | ToLCGHB 4 | Tolecusatellitidae | FR717138 ALCuCMB | 034 | b5 | 1396 | *Ageratum conyzoides* |
| PX352832 | BF48_7_B_4 | Leguema | *Betasatellite solanighanaensequarti* | ToLCGHB 4 | Tolecusatellitidae | FR717138 ALCuCMB | 03 | b5 | 1393 | *Ageratum conyzoides* |
| PX352807 | BF13102020_4_B_2 | Leguema | *Betasatellite sidacutae* | SaAB | Tolecusatellitidae | MK032307 CLCuGeB | 0.265 | b6 | 1369 | *Sida acuta* |
| PX352810 | BF36_10_B_3 | Leguema | *Betasatellite sidacutae* | SaAB | Tolecusatellitidae | MK032308 CLCuGeB | 0.236 | b6 | 1369 | *Sida acuta* |
| PX352811 | BF36_11_B_1 | Leguema | *Betasatellite sidacutae* | SaAB | Tolecusatellitidae | MK032308 CLCuGeB | 0.225 | b6 | 1377 | *Sida acuta* |
| PX352808 | BF36_2_B_2 | Leguema | *Betasatellite sidacutae* | SaAB | Tolecusatellitidae | MK032308 CLCuGeB | 0.26 | b6 | 1370 | *Solanum lycopersicum* |
| PX352809 | BF36_9_B_2 | Leguema | *Betasatellite sidacutae* | SaAB | Tolecusatellitidae | MK032308 CLCuGeB | 0.26 | b6 | 1367 | *Sida acuta* |
| **RCA-RFLP** | |  |  |  |  |  |  |  |  |  |
| PX368861 | BFG727_P6c_16 | Goue | *Colecusatellite gossypigeziraense* | CoLCGA | Alphasatellitidae | MK032297 CoLCGA | 0.038 | a1 | 1379 | *Abelmoschus esculentus* |
| PX368862 | BFG1126_B07a_16 | Goue | *Colecusatellite gossypigeziraense* | CoLCGA | Alphasatellitidae | MK032300 CoLCGA | 0.02 | a1 | 1386 | *Abelmoschus esculentus* |
| PX368867 | BFG1199BL_16 | Goue | *Colecusatellite gossypigeziraense* | CoLCGA | Alphasatellitidae | MK032300 CoLCGA | 0.009 | a1 | 1468 | *Sida acuta* |
| PX368868 | BFG1201BI_16 | Goue | *Colecusatellite gossypigeziraense* | CoLCGA | Alphasatellitidae | MK032301 CoLCGA | 0.001 | a1 | 1465 | *Sida acuta* |
| PX368864 | BFG301BI_16 | Leguema | *Colecusatellite gossypigeziraense* | CoLCGA | Alphasatellitidae | MK032300 CoLCGA | 0.014 | a1 | 1462 | *Sida acuta* |
| PX368865 | BFG301BL_16 | Leguema | *Colecusatellite gossypigeziraense* | CoLCGA | Alphasatellitidae | MK032300 CoLCGA | 0.014 | a1 | 1462 | *Sida acuta* |
| PX368863 | BFG301BM_16 | Leguema | *Colecusatellite gossypigeziraense* | CoLCGA | Alphasatellitidae | MK032300 CoLCGA | 0.014 | a1 | 1466 | *Sida acuta* |
| PX368866 | BFG301BO_16 | Leguema | *Colecusatellite gossypigeziraense* | CoLCGA | Alphasatellitidae | MK032300 CoLCGA | 0.014 | a1 | 1469 | *Sida acuta* |
| PX368870 | BFG983_B19e_16 | Goue | *Colecusatellite gossypigeziraense* | CoLCGA | Alphasatellitidae | MK032297 CoLCGA | 0.009 | a1 | 1386 | *Leucas martinicens* |
| PX368871 | BFG192BJ_16 | Leguema | *Colecusatellite gossypigeziraensecundi* | CoLCGA 2 | Alphasatellitidae | KY652676 CoLCGA | 0.223 | a2 | 1444 | *Sida acuta* |
| PQ623045 | BF5_B14c_16 | Leguema | *Begomovirus solanummaliense* | ToLCMLV | Geminiviridae | MH794643 ToLCMLV | 0.017 | A2 | 2803 | *Solanum lycopersicum* |
| PQ623047 | BF529_B14c_16 | Leguema | *Begomovirus solanummaliense* | ToLCMLV | Geminiviridae | MH794643 ToLCMLV | 0.017 | A2 | 2796 | *Solanum lycopersicum* |
| PQ623046 | BF579_7c_16 | Leguema | *Begomovirus solanummaliense* | ToLCMLV | Geminiviridae | MH794643 ToLCMLV | 0.012 | A2 | 2804 | *Solanum lycopersicum* |
| PQ623041 | BF1163_B12j_16 | Goue | *Begomovirus gossypigeziraense* | CLCuGeV | Geminiviridae | MH794654 CLCuGeV | 0.016 | A7 | 2761 | *Abelmoschus esculentus* |
| PQ623044 | BF301P10b_16 | Leguema | *Begomovirus gossypigeziraense* | CLCuGeV | Geminiviridae | FJ469627 CLCuGeV | 0.019 | A7 | 2755 | *Sida acuta* |
| PQ623043 | BFG192BF_16 | Leguema | *Begomovirus gossypigeziraense* | CLCuGeV | Geminiviridae | MH794664 CLCuGeV | 0.018 | A7 | 2744 | *Sida acuta* |
| PQ623042 | BFG299BI_16 | Leguema | *Begomovirus gossypigeziraense* | CLCuGeV | Geminiviridae | MH794666 CLCuGeV | 0.012 | A7 | 2747 | *Sida acuta* |
| PQ879967 | BFG711EVe1_16 | Goue | *Begomovirus sidafasoense* | SYMBFV | Geminiviridae | JF694462 CleLCrV | 0.206 | B2 | 2759 | *Sida sp* |
| PX368869 | BFG727_P6a-6_16 | Goue | *Betasatellite gossypigeziraense* | CLCuGeB | Tolecusatellitidae | MK032304 CLCuGeB | 0.016 | b1 | 1343 | *Abelmoschus esculentus* |

**Supplementary Table 3: Summary statistics of infection networks in Goué and Léguéma.**

| **Site** | **Nb. of Samples** | **Plant richness** | | **Infection (%)^$^** | **Virus Richness (nb of clusters)^£^** | **Co-infection (%)^£^** | **Host richness^£^** | | **Network^£^** | |
| --- | --- | --- | --- | --- | --- | --- | --- | --- | --- | --- |
|  |  | **Nb of genus** | **Nb of species** |  |  |  | **Nb. of genus** | **Nb. of species** | **Nb. of modules** | **Modularity** |
| Goué | 495 | 15 | 19 | 20.1 | 17 | 58.5% | 7 | 10 | 5 | 0.67 |
| Léguéma | 573 | 15 | 20 | 15.6 | 23 | 63.1% | 9 | 12 | 6 | 0.58 |
| Combined | 1068 | 17 | 25 | 18.1 | 30 | 61.3% | 10 | 15 | 7 | 0.62 |

^$^based on short read sequencing screening

^£^based on full genome sequencing data
